# Challenges and Opportunities for Bayesian Statistics in Proteomics

**DOI:** 10.1021/acs.jproteome.1c00859

**Published:** 2022-03-08

**Authors:** Oliver M. Crook, Chun-wa Chung, Charlotte M. Deane

**Affiliations:** † Department of Statistics, University of Oxford, Oxford OX1 3LB, United Kingdom; ‡ Structural and Biophysical Sciences, GlaxoSmithKline R&D, Stevenage SG1 2NY, United Kingdom

**Keywords:** Bayesian statistics, uncertainty, proteomics, mass spectrometry, phase-separation, workflow

## Abstract

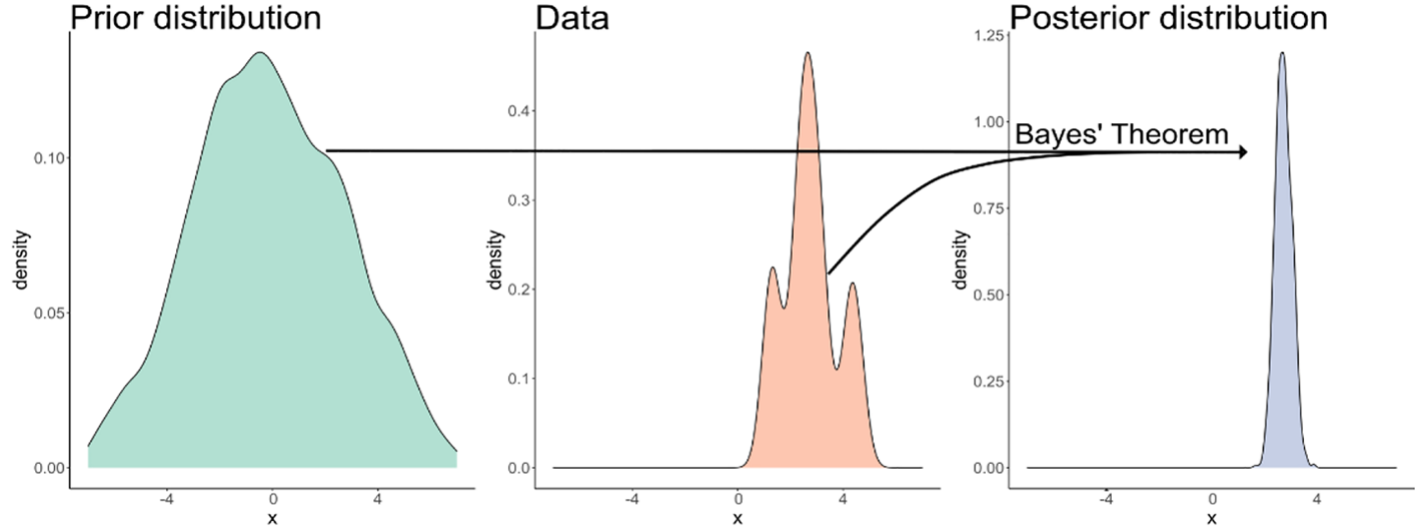

Proteomics is a data-rich science with complex experimental designs and an intricate measurement process. To obtain insights from the large data sets produced, statistical methods, including machine learning, are routinely applied. For a quantity of interest, many of these approaches only produce a point estimate, such as a mean, leaving little room for more nuanced interpretations. By contrast, Bayesian statistics allows quantification of uncertainty through the use of probability distributions. These probability distributions enable scientists to ask complex questions of their proteomics data. Bayesian statistics also offers a modular framework for data analysis by making dependencies between data and parameters explicit. Hence, specifying complex hierarchies of parameter dependencies is straightforward in the Bayesian framework. This allows us to use a statistical methodology which equals, rather than neglects, the sophistication of experimental design and instrumentation present in proteomics. Here, we review Bayesian methods applied to proteomics, demonstrating their potential power, alongside the challenges posed by adopting this new statistical framework. To illustrate our review, we give a walk-through of the development of a Bayesian model for dynamic organic orthogonal phase-separation (OOPS) data.

## 1 Introduction

Decision-making spans the entire research process. Ultimately, it is a choice to believe an explanation for a phenomena given the current evidence. For some theories, the evidence is overwhelming: careful mechanistic experiments and verifiable model predictions have never contradicted that theory.^106^ This scenario is, however, rare. In practice, we make decisions under uncertainty, and the evidence is not clear-cut.^101^ Bayesian statistics allows us to make inferences from that evidence to enable decision-making in those cases.^35^ In contrast to *frequentist* methods, Bayesian inference allows us to use probability to model degrees of belief rather than just frequencies. Consequently, models that are consistent with the available evidence are more probable, and incompatible models are less probable.^37,87^ By using probability theory in this manner, there is a recipe for taking *prior beliefs* (i.e., information encoded by domain expertise) and updating them to *posterior beliefs* using observed data.^35^ This posterior probability distribution quantifies the models compatible with domain expertise and our experimental data.^37^ This recipe is known more formally as *Bayes’ theorem*.

Mass-spectrometry-based proteomics is a complex scientific field.^2^ The technique’s versatility allows it to explore differential abundance,^2^ protein turnover,^66^ interactions,^46^ thermal stability,^65^ structure,^89,64^ spatial information,^38,33,3^ and more.^99,74,48^ In each case, data are manipulated, thresholded, and filtered so that a statistical test or machine learning algorithm can be applied. The results are then frequently summarized as a single value, which is granted the role of arbiter of truth. Bayesian statistics allows the propagation or quantification of uncertainty in all of the step of an analysis, replacing the current implicit ad hoc approaches with explicit models and summarizing the output with a probability distribution consistent with the data.^37^ This paradigm progression provides not only an ability to ask new questions of the data but also a consistent way to perform inference and criticize models.^35,37^

Bayesian statistics offers considerable potential for the examination of proteomics data; despite this, it has not been readily adopted in the community. Here, we review the contribution that Bayesian statistics has already made to proteomics, clarify the Bayesian workflow and how it can be applied, highlight a number of modeling strategies, and outline current challenges for the proteomics community. Throughout, we illustrate our analysis with examples from the proteomics literature, focusing on building a model for dynamic organic orthogonal phase separation (OOPS) data.^80^ This structure is designed to inform a number of readers including those unfamiliar with Bayesian statistics and those developing tools to analyze proteomic data.

## 2 Main

### 2.1 Bayes, in Brief

**Figure 1:**
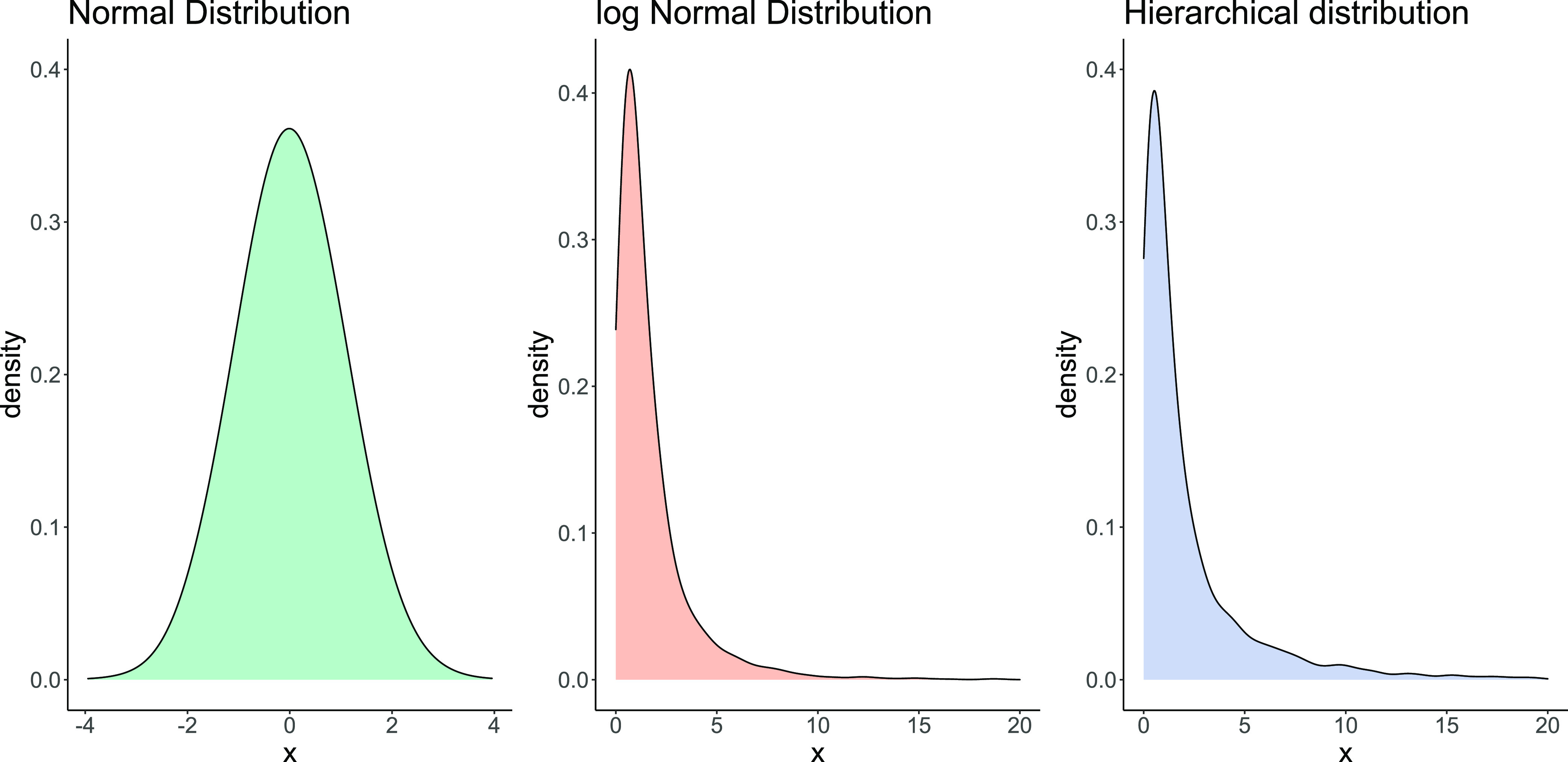
Example probability distributions. The normal distribution (left) and log-normal distribution (center) with mean and log-mean parameter 0 and variance and log-variance parameter 1. (right) Hierarchical distribution defined by a log-normal distribution with log-variance 1 and log-mean distributed as a normal distribution with mean 0 and variance 1.

Before reviewing the contributions that Bayesian statistics has already made to proteomics, we introduce the technical background and notation. We use *P*(*E*) to denote the probability of the event *E*. *E* can be anything from “it rains tomorrow” to “my parameter falls between the values *a* and *b*”. We let *D* be notation for the observed data, for example, from a shotgun proteomics experiment and let *x* be a data point from *D*, such as a measurement for a particular protein. We assume that *x* is a sample from some probability distribution *p* and we write *x* ∼ *p*(*x*|θ), for example this could be a log-normal distribution (see Figure 1). A *sample* is a random draw from that probability distribution, which can be thought of as picking out a number from a bag where the probability of selecting that number is defined by the probability distribution. The notation *p*(*x*|θ) describes the probability distribution, where *x* is the variable. The vertical bar means “given”, so that everything on the right-hand side is assumed to be known. For example, θ for a normal distribution is the mean and variance.

Let α be hyperparameters of the parameter distribution, such that the θ themselves are drawn from a probability distribution θ ∼ *p*(θ|α). For example, the log-mean parameter of the log-normal distribution could be drawn from a normal distribution. The log-normal distribution has two parameters, the log-mean *m* and log-variance σ^2^, which are not equal to the mean and variance of the distribution. In the case of the log-normal distribution, we have θ = (*m*, σ^2^) and note that *m* is any number, while σ must be positive. From here, we can define a probability distribution for *m* by letting  and hence α = (μ, ν^2^). In Figure 1, we plot the distribution defined by ν^2^ = σ^2^ = 1 and μ = 0, which we can see has higher mean and variance than the log-normal distribution with log-mean parameter 0 and log-variance parameter 1. Such *hierarchical distributions* are useful for describing scenarios where the probabilities depend on levels of information. For example, we could define the probability distribution of it raining today given that it is cloudy: *p*(rain|cloudy today) = 0.8 and *p*(rain|not cloudy today) = 0.1. This distribution depends on the parameter cloudy or not, which also occurs with some probability; i.e., the probability that it is cloudy today given that it was cloudy yesterday could be given by *p*(cloudy today|cloudy yesterday) = 0.3. The flexibility of hierarchical distributions allows us to model an array of scenarios.

The *prior distribution* captures our domain expertise and is the distribution of the parameters before any data is observed: *p*(θ|α). The *prior* could capture, say, that abundance values are positive and are unlikely to exceed the number of grains of sand on Earth. The sampling distribution is the distribution of the data given the parameters *p*(*D*|θ), we can write this as a function *L*(θ|*D*) called the *likelihood*. If we average (or *marginalize*) the distribution of the data over the parameters, we obtain the so-called marginal likelihood:
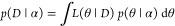
1

We can see that the marginal likelihood is the likelihood multiplied (or weighted) by the prior and then integrated over the parameters. The symbol ∫ denotes integration, an operation which assigns numbers to functions. For example, the area under a curve is given by the integral of the function describing that curve. Integration arises in probability because we can colloquially think of probability as being the “area” of an event on a distribution. The posterior distribution is the distribution of the parameters after having observed data and is determined by Bayes’ theorem, as the following:
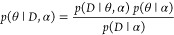
2

Bayes’ theorem tells us the mathematical way to update beliefs in light of evidence: simply multiply our prior and likelihood and renormalize by the marginal likelihood. Returning to our weather example, we can demonstrate an application of Bayes’ theorem. Given that it has rained today and it was cloudy yesterday, what was the probability that it was cloudy today? Formally, we are being asked for
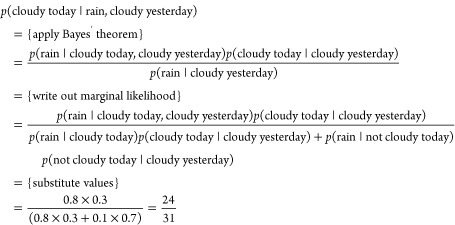
3

Bayes’ theorem implies a self-consistency property. The posterior averaged over the data returns the prior:^95^
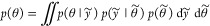
4where *ỹ* ∼ *p*(*D*|θ̃). This can also be thought of in terms of simulation. First, sample parameter values from the prior distribution of the parameters. Then, sample data from the model given these parameter values. Then, given these data, sample from the posterior distribution of the parameters. These samples will also be distributed according to the prior distribution. In Bayesian analysis, to perform prediction, instead of simply taking a single parameter value forward, we use averaging, which results in a distribution of values:
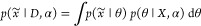
5

In summary, Bayesian statistics provides us with a distribution of plausible parameter values from the *posterior* and a distribution of hypothetical predicted values from the *posterior predictive distribution*. It is these distributions that quantify uncertainty in a Bayesian analysis. We can then ask bespoke question of these probability distributions; for example, *P*(θ > 2|*D*, α) = ∫_2_^∞^*p*(θ|*Dα*) dθ is the probability that a parameter is greater than 2. For proteomics, these could be the probability that a fold-change exceeded a certain value or the probability that a spectrum belongs to a particular peptide.

### 2.2 Bayesian Contributions to Proteomics

#### 2.2.1 Bottom-Up Proteomics and Differential Abundance

Next, we discuss Bayesian approaches already applied to proteomics. We focus on proteomics data generated via mass spectrometry but refer to contributions for the reverse-phase-protein-array (RPPA) literature^22,73,61^ and 2D gel electrophoresis.^71^ These fields are typically interested in testing for differences between biological samples due to a perturbation of interest. Using Bayesian statistics, one could model these changes and quantify differences using the probability of a particular fold-change. A number of approaches have been aimed at quantification and differential abundance analysis of bottom-up proteomics data.^79,98,97,86,78,68,91,75,90,13^ Carvalho et al.^13^ describe the use of Bayesian statistics to improve the analysis of spectral counting data using a Poisson likelihood and calculating the probability of detecting a protein in a particular sample. However, they do not exploit the full Bayesian toolkit and simply interpret their probabilities as *p*-values. Thus, it is not clear whether this is any better than simply using a likelihood-based method. A number of approaches^98,97,78,90^ argue for propagating and quantifying the uncertainty in the analysis rather than simply the underlying quantitation values, through either relaxing the parsimony assumption,^90^ including ion statistics via a two-level Beta-Binomial model,^78^ or jointly modeling identification and quantitation statistics.^97,98^ Millikin et al.^68^ use an analogue of the *t* test and exploit the posterior distribution by using an interval thresholding approach. O’Brien et al.^75^ note the inherent compositional nature of labeled proteomics approaches and include a modeling parameter to model ratio compression allowing a better estimation of the true fold-changes. Importantly, this parameter is shared across all proteins, which allows them to estimate the parameter accurately even for proteins with few observed peptides. Jow et al.^49^ also model isobaric labelled mass-spectrometry data but do not model ratio compression. Meanwhile, for label-free experiments, O’Brien et al.^76^ explicitly model missingness showing that jointly modeling missingness and abundance leads to improved performance. All of these approaches demonstrate some benefit over previously applied methods, suggesting that combining these methods would provide further improvements. This also suggests that translating these methods to other proteomics techniques would be a fruitful endeavor.

#### 2.2.2 Protein and Peptide Identification

One of the fundamental problems in mass-spectrometry-based proteomics is identifying a peptide from spectra. Spectra can be very noisy, and *b*^–^, *y*^–^ ions can be missing which results in a complex observation process. Furthermore, we have prior knowledge of observing particular amino acid sequences and knowledge of the cleavage process. This is an ideal scenario for the application of Bayesian methods. These methods could model spectra directly and report the probability that a particular amino acid belongs to a particular spectrum. Indeed, a number of approaches have been applied.^15,42,55,19^ Chen et al.^15^ used a fairly simple framework to calculate peptide identification probabilities based on peptide coordinates. Halloran et al.^42^ employed a dynamic Bayesian network in a method called DRIP, allowing for insertions and deletions to the spectra. By modeling possible alignments between theoretical and observed spectra, they were able to calculate the most probable peptide match. The authors found that their approach was an improvement over available methods particularly for low-resolution MS2 data. Lewis et al.^55^ took a different approach to the same problem, incorporating a scoring function into a likelihood model. Their model also allowed deletions directly via indicator functions. Insertions were characterized by excessive deviations from the spectra of the candidate peptide, where excessive is characterized probabilistically via Laplace noise. The authors also included prior information about possible cleavage pairs as well as prior information about the probability of observing a particular peptide sequence in the data set. Finally, in contrast to Halloran et al.,^42^ they made full use of Bayesian methods and provide a posterior distribution over possible peptides and parameters. This could have allowed multiple peptides to be associated with a spectrum with differing certainty which could have been used in downstream analysis. Claassen et al.^19^ tackled a slightly different problem and used a nonparametric Bayesian model to predict the coverage in sequential LC-MS/MS experiments but suggest that their approach could also have been adapted to database searching and de novo sequencing. Again, these approaches are all shown to have benefits over the previously applied methodology. The clearest is the inclusion of more information and the ability to provide a flexible, and well-rationalized model to the underlying data. The ability to exploit uncertainty captured by the posterior distribution for downstream analysis is far more insightful than simply point estimates from a Bayesian analysis. However, these approaches have not yet been widely adopted by the community. This could be because the methods are difficult or expensive to apply, or the benefits are not compelling. We aim to show, throughout this Review, that benefits of a Bayesian analysis are compelling and straightforward to obtain.

#### 2.2.3 Proteoforms and Post-Translation Modifications

A number of approaches are interested in applications to proteoform analysis (splice isoforms) or post-translational modifications,^18,105,57,93,62^ and Bayesian modeling in these fields could answer whether a peptide was modified or not and the localization of that modification. Chung et al.^18^ employed a nonparametric mixture model to jointly model the modification mass for each PTM group and the true (unobserved) location of the modified amino acid. Their approach outperformed other approaches, is fully automated, and provides modification confidence scores. However, the approach did not model the underlying spectrum, which could have resulted in unnecessary false positives. Webb-Robertson et al.^105^ tackled the proteoform problem by deconvolving peptides into signatures even if they are associated with the same protein. However, they only used a Bayesian point estimate rather than exploiting the full posterior distribution. Lim et al.^57^ used a Bayesian model to estimate the phosphorylation stoichiometry using Bayesian statistics. By incorporating a physically plausible model, they removed problems with previous models that could have allowed negative stoichiometry. Their joint model allowed them to borrow power across replicates, and they reported downstream uncertainty. Shteynberg et al.^93^ use a Bayesian mixture model to compute probabilities for modification sites. This allowed them to combine precomputed scores in a rational way, but again, they did not examine the full posterior distributions. Mallikarjun et al.^62^ employed a Bayesian linear regression modeling strategy to analyze differential PTM data, suggesting that their approach outperformed other methods and could allow uncertainty in missing values. The main benefit here appeared to be the regularization of the parameters using priors rather than specifically the uncertainty quantification in the analysis.

#### 2.2.4 Biomarkers and Clinical Proteomics

Protein biomarkers, molecular indicators of aberrant processes or disease, and clinical proteomics are a key component of proteomics research. For a review of Bayesian method development in biomarker discovery, see Hernández et al.^44^ In these fields, Bayesian statistics can simultaneously model protein abundance levels and the contribution of exogenous variables, allowing researchers to disentangle disease related variability versus variability due to environmental factors. Morris et al.^69,70^ developed Bayesian wavelet-based functional mixed models for mass-spectrometry-based proteomics data. Their advanced framework allowed the simultaneous use of nonparametric fixed and random effects, which facilitated an adjustment for clinical and experimental covariates that could affect the intensity and location of spectra. Working with posterior distributions, they were able to compute important quantities such as the probability of intensity changes for fixed fold levels and were able to control a Bayesian false discovery rate; that is, the posterior probabilities are thresholded to control the error rate. Liao et al.^56^ combined that framework with image analysis methods to enable biomarker discovery from LC-MS data. Hwang et al.^47^ developed a pipeline, MS-BID, for biomarker analysis that uses a Bayesian analysis of variance (ANOVA). Harris et al.^43^ applied a Bayesian hierarchical linear probit regression (regression where only two outcomes are allowed) model to determine discriminative biomarkers from mass-spectrometry data. They found that their approach improved over a simple *K*-nearest neighbor method. Furthermore, by using posterior probabilities, they were able to determine which samples will be the most promising for prognostics. Kuschner et al.^52^ demonstrated a Bayesian network to perform feature selection from mass-spectrometry data. The selected features then provided excellent predictive power. Though again this approach still used a Bayesian point estimate instead of full posterior distributions. Deng et al.^27^ developed a Bayesian network which allows them to integrate mass-spectrometry and microarray data, allowing them to borrow power between mRNA and protein levels. Here, they made use of the flexibility of Bayesian statistics to incorporate different modalities and weigh up the uncertainty between different data sets. More recently Liu et al.^58^ developed Bayesian function-on-scalar quantile regression for mass-spectrometry data. This approach noted that biomarker difference may not be apparent at mean regression but rather at a particular quantile (such as the 0.95 quantile). It simultaneously accounted for the functional nature of MALDI-TOF data and incorporated prior knowledge for adaptive regularization and a basis representation which allowed borrowing of power. They found that their method identifies biomarkers overlooked by mean regression.

Bayesian methods for biomarker and clinical proteomics are more developed than other examined proteomics subfields with several exemplary methods that make full use of the flexibility of Bayesian modeling and the rich output of the posterior distribution.

#### 2.2.5 Chromatography

To facilitate peptide identification in mass spectrometry, a liquid-chromatography step is usually applied. The time at which a peptide elutes from the liquid chromatography, called the retention time, can be used as additional information to help identify peptides. However, there is uncertainty in this retention time, and it can vary from one run to another. Bayesian models could capture the uncertainty in these data, which could be better used to align data between runs. Chen et al.^14^ developed a Bayesian model called DART-ID that models a latent (unobserved) global retention time alignment. This alignment allowed them to combine the outputted posterior error probability of MaxQuant with the inferred RT density in each experiment. Hence, by using this result, they updated their confidences and improved coverage in experiments by 50%. While their approach is powerful, they only used a point estimate and obtained uncertainty through bootstrapping. Maboudi Afkham et al.^60^ are interested in the uncertainty in peptide retention time measurements. Using a Gaussian process regression method, they were able to accurately predict retention times and obtain uncertainty estimates. They then used the posterior distribution from the regression analysis as a variable retention time window to identify potentially incorrect peptides. This improved over fixed windowing strategies. One potential strategy for improvement, in a similar vein to DART-ID, would have been to update the identification probabilities based on the deviation probability from the predicted retention time. This approach naturally fits within a Bayesian framework.

#### 2.2.6 Intact, Top-Down, and Structural Proteomics

Bayesian statistics could have a number of uses in the fields of intact, top-down, and structural proteomics. For example, an ensemble of structures concordant with the data, along with their relative probabilities, could be reported rather than simply a single estimate. Saltzberg et al.^84^ proposed a Bayesian model to resolve residue-level information from hydrogen–deuterium exchange mass spectrometry. They chose uninformative priors, and though they performed inference using Monte Carlo methods, they did not use the posterior distribution. Furthermore, they do not justify why their model allows for negative deuterium incorporation, which maybe arises from a misunderstanding of the positivity constraint induced by their exponential likelihood model. Proteoform analysis is one of the key challenges in top-down proteomics. LeDuc et al.^54^ introduced a *C*-score, not to be confused with a *C*-statistic, to facilitate automated identification and characterization of proteoforms from top-down proteomics data. Ultimately, their approach allowed them to rank probable proteoforms having observed their data. Performing an analysis in a Bayesian framework allowed them to specify a generative model, provide expert prior information, and carefully model the underlying noise distribution. Their proposed *C*-score is essentially a transformed posterior error probability. However, despite their Bayesian framework, they opted for a point estimate of their model, which could have been greatly enhanced by examining the full posterior of their model. A point estimate of a probability distribution is a good way to summarize it visually. However, it does not completely characterize that distribution; for example, summarizing a normal distribution by its mean is insufficient to describe it. For complex, high-dimensional distributions, it is often challenging to find a single visual representation because diagrams can usually only represent three quantities simultaneously. One way to represent uncertainty in this problem is to display the probability of one amino acid proceeding another using a 20 × 20 matrix, where each entry of the matrix is a probability of observing that ordered pair of amino acids (see Figure S1). This matrix can also be expanded to represent modifications or noncanonical amino acids. A more direct representation of a probability distribution of a spectrum is to use a contour plot in the *m*/*z*–intensity coordinates. Here, the *z* dimension corresponds to the probability of observing that *m*/*z*–intensity pair. An example for the MS1 spectra of PARRDAARA is visualized in Figures S2 and S3. Marty et al.^63^ proposed a Bayesian deconvolution algorithm for ion mobility spectra, which was extended by Kostelic et al.^51^ Their approach allowed the convolution of the charge distribution with the peak shape to obtain a flexible deconvolution approach. The wide extent of their applications demonstrated the clear benefits of their method. However, their approach also used a point estimate from their analysis. Hence, apart from the use of prior information, it is not clear what particular benefit a Bayesian analysis had for their approach.

#### 2.2.7 Functional Proteomics

Functional proteomics methods aim to decipher protein function on a system-wide scale. Indeed, Bayesian statistics could be used to quantify the probability of two or more proteins interacting. One approach is spatial subcellular proteomics^33,17^ where proteins are localized to their subcellular niche using mass-spectrometry data. Bayesian approaches have been developed for biochemical fractionation-based subcellular proteomics.^20,22,21,23,24^ Crook et al.^20,22,21^ demonstrated that Bayesian modeling can quantify uncertainty in protein subcellular localization and identify cases where this may correspond to multilocalizing proteins. Crook et al.^20^ showed that even a Bayesian point estimate may overlook these cases, and more information is obtained by examining the full posterior distribution. Crook et al.^23^ allowed the uncertainty in the number of subcellular niches to be accounted for and showed that allowing additional niches can be uncovered. However, the model appeared sensitive to the prior choices and should be chosen carefully. Crook et al.^24^ built on these experiments to analyze differential localization experiments showing that modeling uncertainty improved power and interpretation compared with other methods. This fully Bayesian analysis, however, is computationally intensive as it attempts to model many data sets at once. Another functional approach is affinity purification mass spectrometry (AP-MS), which allows us to determine protein interactions and complexes.^17^ Choi et al.^16^ developed a nonparametric Bayesian model to bicluster AP-MS data. They sampled from the posterior distribution and are hence able to report the uncertainty in the clustering. However, their nested model assumed that the conditional on the Bait cluster and the Prey clusters are independent, and their model assumed exchangeability (permutation leads to the same probability distribution) of the rows and columns. Fang et al.^28^ proposed a semiparametric model for thermal protein profiling after identifying proteins that deviate from classic sigmoid behavior. Semiparametric models combine interpretable parametric models with more flexible nonparametric models. Using a Bayesian analysis, they critically assessed the semiparametric and parametric model fits and demonstrate that those proteins that are better modeled by the semiparametric model share functional enrichments. Again, this fully Bayesian approach had demanding computational requirements, which may explain why many methods choose not to employ Bayesian methods.

### 2.3 Bayesian Workflow

#### 2.3.1 Motivating Example

**Figure 2:**
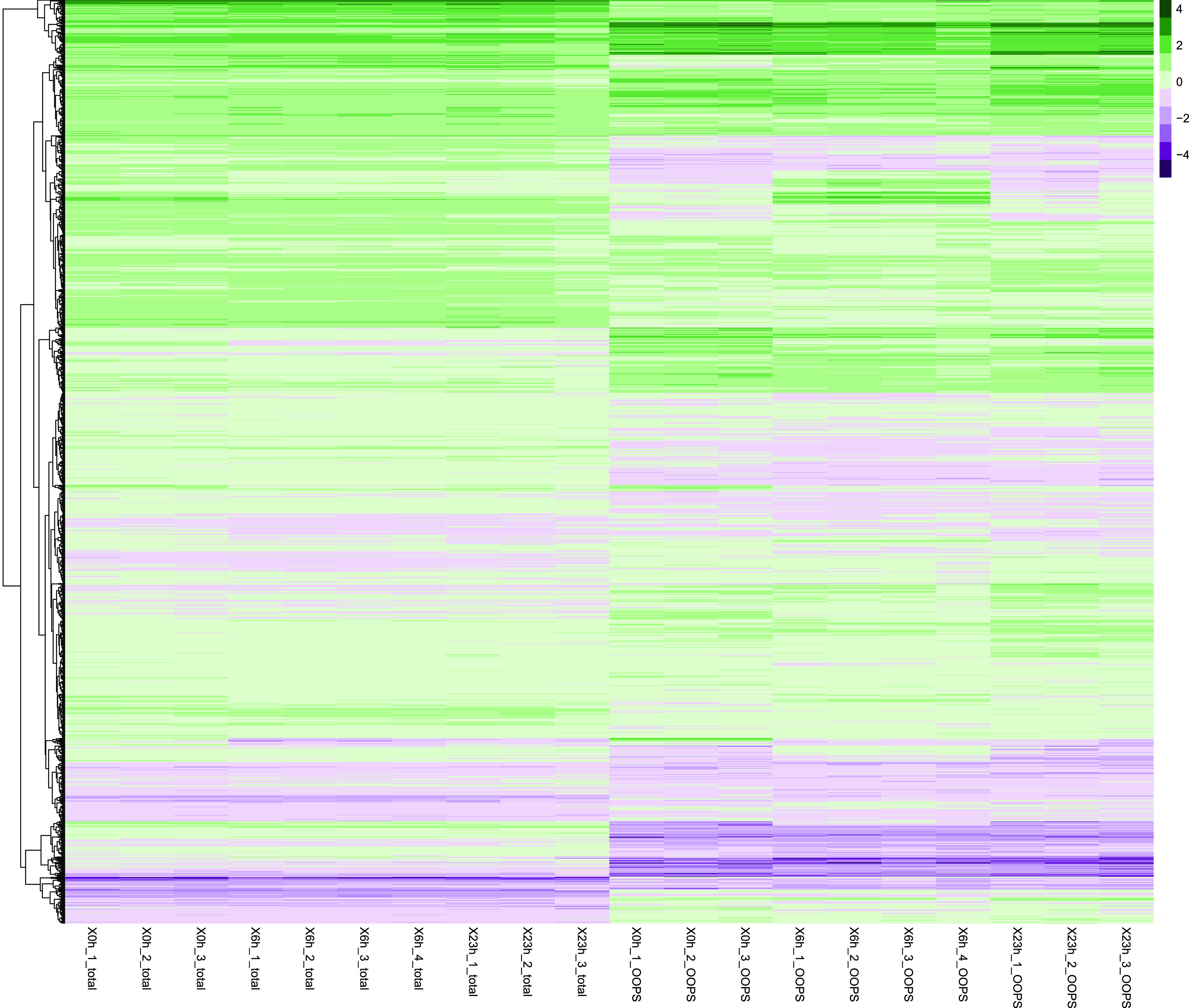
Exploratory data analysis of OOPS. Heatmap of the mass-spectrometry data generated by the OOPS experiment. The tree clustering is produced using Ward’s method. Each cell represents *z*-score normalized protein abundances. Column annotations are encoded as *X*time_replicate number_sample type.

To illustrate the Bayesian workflow, we examine some recently introduced proteomics data generated using the orthogonal organic phase separation (OOPS) method of Queiroz et al.^80^ This method is able to efficiently enrich for RNA-binding proteins and, hence, by adapting to the dynamic setting, is able to examine differential RNA binding. This is where the proportion of a particular protein bound to RNA changes depending on the condition. Here, we examine an experiment where thymidine-nocodazole was used to induce cellular arrest. Total and OOPS-enriched protein abundances where obtained at 0, 6, and 23 h after treatment. Each experiment was performed in triplicate, except for at 6 h when four replicates were taken. The 10 total and 10 OOPS samples were labeled using 2 separate TMT 10-plex kits, and quantitative mass spectrometry was performed in two runs using SPS-MS3 on an Orbitrap Fusion Lumos instrument. Here, we attempt to use the Bayesian toolkit to model these data and answer questions about changes in RNA binding. A heatmap of the data is shown in Figure 2. A protein was chosen at random to illustrate the modeling process: NCAPD2, a regulatory subunit of the condensin complex. NCAPD2 is known to have differential subcellular localization throughout the cell cycle.^88^

#### 2.3.2 Generative Modeling

Having highlighted the successes and limitations of some of the contributions of Bayesian methods to mass-spectrometry-based proteomics, below, we outline the Bayesian workflow to facilitate it for proteomics. The first tension of Bayesian analysis is the pairing of the likelihood (the model of the data) and the prior (the model of the parameters of the likelihood).^36,37,7^ On one hand, the word *prior* suggests that it must be chosen first; however, without knowledge of the likelihood, it makes little sense to start selecting priors—we may not even know the parameters of the model. Thinking of the likelihood and prior as a pair reduces this conceptual tension. It also leads to an explicit way to check our modeling assumptions via generative and predictive modeling.^7^ A generative model generates data consistent with the data. In the same way that a cake recipe, when the steps are followed, generates a cake. The prior has good predictive properties if the *posterior predictive distribution* can predict new data generated from similar experiments. To be explicit, given a likelihood and prior, we can simulate data *y*. First, sample the parameters of the likelihood, *L*(θ|*D*), from the prior, *p*(θ|α), and then, given these parameters, sample data from the model:
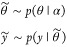
6

This leads us to define the *prior predictive distribution*:
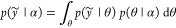
7

For example, assume that data are modeled as a log-normal distribution with parameter *m* and σ = 1. Assume that the prior on *m* is a normal distribution with mean 0 and variance 1. The prior predictive distribution first samples *m*_*i*_ from  and then log *y*_*i*_ ∼ *N*(*m*_*i*_, 1). In fact, this is the hierarchical distribution shown in Figure 1. There are a number of key observations. First, the prior predictive distribution has no knowledge of the data, aside from the modeling assumptions of the domain expert. Second, the likelihood and prior are now explicitly coupled, and so poor modeling choices in either the likelihood or prior will be apparent via the prior predictive. Third, the failure of uniform or uninformative priors as a default is clear, as they will generate unrealistic data.

In our OOPS example, we model log protein abundance as a linear model of sample type (whether total or OOPS) and time (0, 6, and 23 h). Since we are interested in changes in the proportion of protein bound to RNA, we include an interaction effect between time and sample type. We then use Gaussian priors on the coefficients of the effects and an exponential prior on the standard deviation of the Gaussian noise. Formally, the model can be written as
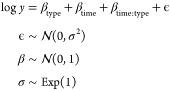
8

**Figure 3:**
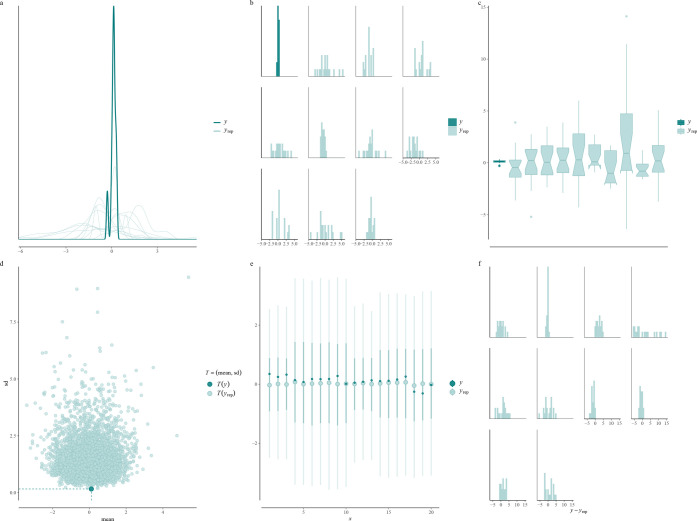
Prior predictive checks. Prior predictive checks applied to OOPS data. *y* denotes the observed data, while *y*_rep_ denotes the simulated data from the prior predictive distribution. (a) Kernel density estimation-based checks. (b) Histogram-based checks. (c) Box-plot-based checks. (d) Summary statistics checks. (e) Interval-plot-based checks. (f) Error histogram-based checks. This figure can be reproduced in the vignette in the Supporting Information and evaluated for other prior choices.

The priors were chosen arbitrarily, and we can use a prior predictive check to see whether this leads to a sensible generative model. Figure 3 show a variety of prior predictive checks using different summaries of our observed and simulated data. We see that the our generative model is too diffuse compared with the observed data and produces large deviations beyond what we would expect from a typical proteomics data set. Hence, it is necessary to explore more prior choices using prior predictive checks. In the accompanying vignette in the Supporting Information, we show that our inferences can be better calibrated using an exponential prior with rate 4, which corresponds to 1 in 5000 proteins having a standard deviation in their log abundance above 2.5.

#### 2.3.3 Predictive Modeling

Once our prior and likelihood have seen the data, *D*, they are updated into the posterior distribution. We can then sample new data by first sampling parameters from the posterior distribution and then again sampling from the likelihood:
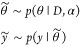
9

This leads to the definition of the posterior predictive distribution:
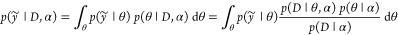
10

We have expanded the integrand using Bayes’ theorem to make a key point explicit: the posterior predictive distribution depends on the likelihood, the prior, and the data. This coupling allows us to make a number of observations. A good choice of prior and likelihood leads to good predictive performance, and overfitting can be examined via the posterior predictive distribution.

**Figure 4:**
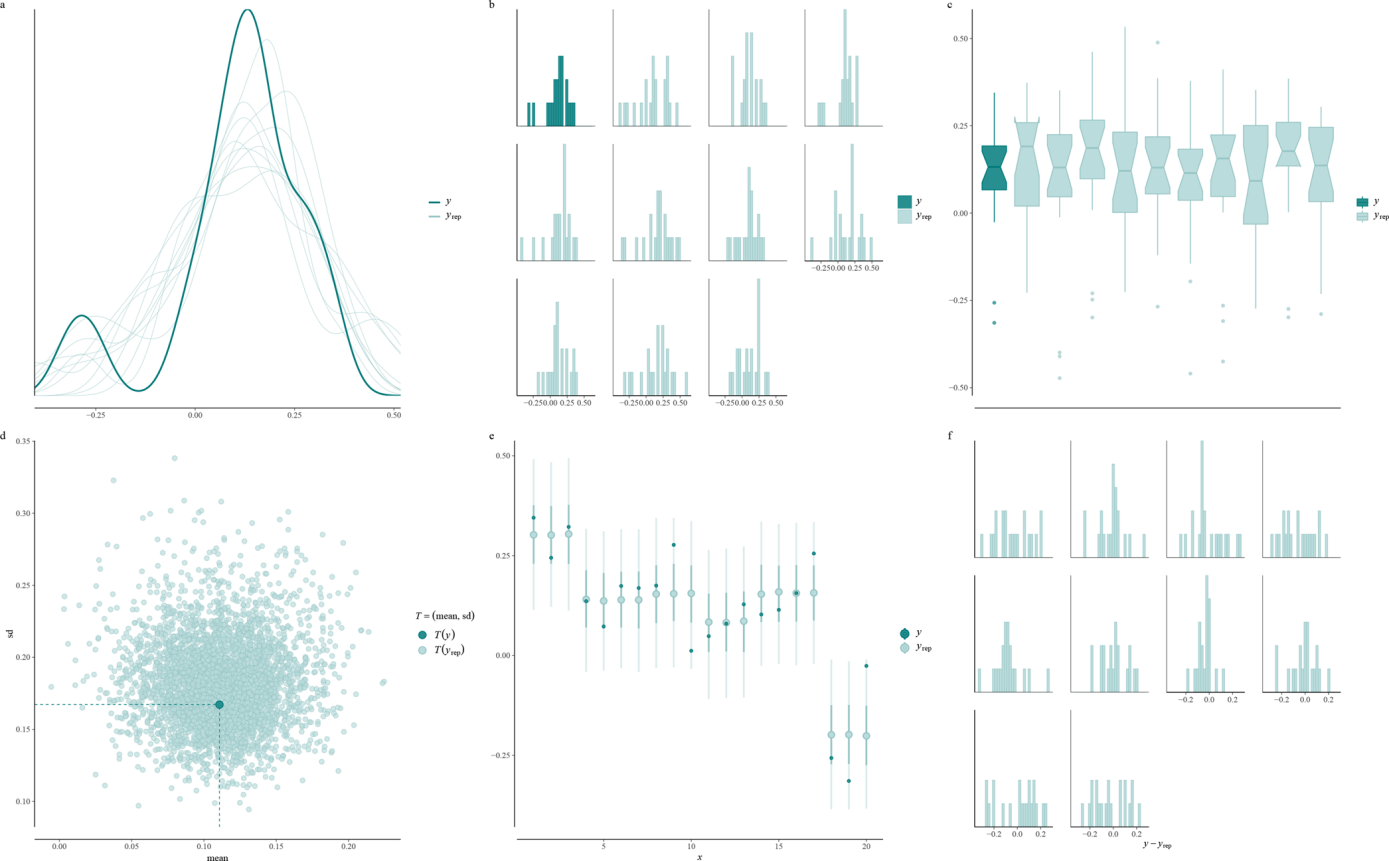
Posterior predictive checks. Posterior predictive checks applied to OOPS data. *y* denotes the observed data, while *y*_rep_ denotes the simulated data from the prior predictive distribution. (a) Kernel density estimation-based checks. (b) Histogram-based checks. (c) Box-plot-based checks. (d) Summary statistics checks. (e) Interval plot-based checks. (f) Error histogram-based checks. This figure can be reproduced using the vignette in the Supporting Information, and more choices can be explored.

Having fitted the model to the data, we can perform a posterior predictive check on our inferences. Figure 4 shows a number of posterior predictive checks, and it also shows that clearly the model has learnt from the data. Visualization shows that samples from the posterior predictive distribution look similar to the observed data. Contrast this with prior predictive checks where the samples from the distribution were very diffuse.

#### 2.3.4 Fitting a Model: Bayesian Computation

In practice, the integrals and probability distribution required for sufficiently flexible modeling are intractable. We can perform inference in a wide array of models using Markov-chain Monte Carlo (MCMC) methods:^39,10^ including Gibbs sampling,^94,34^ Metropolis sampling,^82^ and Hamiltonian Monte Carlo.^45^ Bayesian inference can also be performed using sequential Monte Carlo^26^ or variational inference.^9^ Although the latter can provide a fast approximation of the posterior distribution, it can be arbitrarily inaccurate. Here, we focus on Hamiltonian Monte Carlo, as it forms the basis of modern probabilistic programming languages.^12^

Initially, when an MCMC algorithm begins, it will “move” toward the posterior distribution producing a “sample” at each iteration. An initial warm-up or burn-in section is require to remove bias due to a dependence of the algorithm’s starting values and to adapt some of the algorithm’s tuning parameters to provide efficient inference. Once the warm-up section is complete, there is a sampling period which is run until multiple chains have mixed (sampling from the same posterior distribution). One measure of mixing chains is *R̂*, which is essentially a measure of between and within chain variance.^104^ Current standard practice is that *R̂* should be close to 1. It is also recommended to visualize trace plots and rank histograms for samples from an MCMC algorithm.^31^ Some tools include further diagnostic checks such as divergences, but this is beyond the scope of this Review.^6^Table 1 highlights some probabilistic programming languages that can be used to fit general purpose Bayesian models. For our OOPS example, Bayesian computations are reliable; see the accompanying vignette in the Supporting Information.

**Table 1:** General Purpose Probabilistic Programming Languages^a^

Packages for Bayesian Computation			
computational tool	language	inference method	ref
stan	C++	HMC variant	(12)
brms	R	HMC variant	(11)
MCMCglmm	R	metropolis/slice sampling	(41)
PyMC3	Python	HMC variant	(85)
BUGS	BUGS/R	Gibbs sampling	(59)
Edward	Python	various including variational inference	(100)
Pyro	Python	HMC variant	(8)
Turing.jl	Julia	various including HMC	(32)

a A variety of probabilistic programming languages are available in several languages using modern and efficient inference methods. Amongst these languages, one can fit the vast majority of models used in practice.

#### 2.3.5 Posterior *z*-Scores and Contraction

It is often desirable to evaluate the behavior of a model and whether any model assumptions are preventing us from making sensible inferences. The *posterior z-score* and *posterior contraction* are useful metrics to identify several problems with a model.^7^ Let us assume that we have access to a parameter, θ*, of the true data generating process. The *posterior z-score* for a parameter is defined as
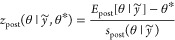
11where *E*_post_ denotes the expectation under the posterior and *s*_post_ the standard deviation under the posterior. The *posterior contraction* is defined as
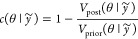
12where *V*_post/prior_ denotes the variance under the posterior/prior. Together, these quantities tell us about how the posterior is learning from the data. If the posterior *z*-score is large, and the posterior contraction is small, then the prior modeling conflicts with the true process—we are unable to learn the true parameter well. If the posterior *z*-score is large, and the posterior contraction is close to 1, this suggest that we are concentrating on an incorrect part of the probability space, and so the model is overfitting. If the posterior *z*-score is small, and the posterior contraction is also small, then the model is poorly informed by the data. The ideal scenario is that posterior contractions are close to 1 and that posterior *z*-scores are close to 0. This tells us that the data are highly informative, and the prior was not biased away from the data generating mechanism. Examples of posterior contractions are shown in the accompanying vignette in the Supporting Information.

#### 2.3.6 Model Selection and Averaging

Using probability allows us to select between competing models that may generate the data. For example, was this model generated by data with two or three clusters/groups? Given two models,  and , we can ask for the  for *i* = 1, 2. The relative plausibility of two models is referred to as the *Bayes factor*,^50^
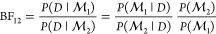
13

The Bayes factor allows an interpretable and quantitative way to evaluate the relative plausibility of two models by examining the ratio of the probabilities of the model generating the data. The Bayes factor is the ratio of the posterior probabilities of the models multiplied by the ratio of the prior probabilities of the models. As such, it incorporates the plausibility of the models before we see any data, with the plausibility of the data after we have seen the data. From a brief calculation, we can see that
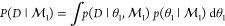
14where θ_1_ are parameters that parametrize model . Here, we see the dependence of the Bayes factor on the prior, and the implicit assumption that we are evaluating models on their prior predictive performance becomes explicit. Thus, using improper/uninformative priors with the Bayes factor would be inappropriate. However, there are further complexities; the most concerning perhaps is that one can inflate the Bayes factor by simply choosing a prior that places probability on unrealistic parts of the parameter space. Typically, a uniform prior would have such an effect. Thus, if you are unsure of the veracity of your prior choices, model evaluation may be better using functions of the posterior predictive distributions.^7^

We have already seen that one of the key mechanics of Bayesian statistics is the ability to average over quantities, rather than simply taking the best parameters forward. This can also be performed with models using so-called Bayesian model averaging.^81^ Let ϕ be a quantity of interest (such as the abundance of a protein from an experiment), and given models  (such as different plausible regression models), we may average them:
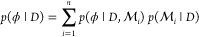
15

This is the average of the posterior predictive distribution for ϕ under the models considered, weighted by their posterior model probability. If we are interested in the Bayesian model average estimate of a particular parameter, we can compute
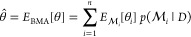
16

Given the sensitivity of the Bayes factor to the prior, it is sometimes useful to consider model selection based on the posterior predictive distribution. One example is the log pointwise predictive density (lpd):^103^
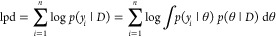
17

This is a measure of the total probability of the observed data as if it were being predicted from having fitted a Bayesian model. Furthermore, it is frequently useful to consider an out-of-sample predictive fit via leave-one-out (LOO) cross-validation:^103^
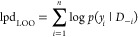
18where *D*_–*i*_ is data without data point *i*. This quantity can be efficiently approximated using the LOO package.^103^ This is the same as the above quantity except that we have hidden *y*_*i*_ from the model, and so it measures the ability of the model to generalize to unseen data. We note that the above definitions can be adapted to any utility or loss function so that the metric of interest can be characterized.

Returning to our OOPS example, in our second vignette in the Supporting Information, we develop more complex models of the data. These models include group-level (random) effects for the replicate number and TMT tag used (see the Modeling Strategies section). Our OOPS example is designed as a typical proteomics experiment where the time points include three or four biological replicates. Proteomics experiments usually have nested hierarchical levels of replicates: for example, biological or technical/injection replicates. Estimating the variability associated with replication typically improves power to detect differences between samples. Including such effects is typically straightforward in a Bayesian analysis through the use of group-level parameters as highlighted in our example below. All of the software packages in Table 1 can be used to construct such models. The three competing models are as follows:Model 1
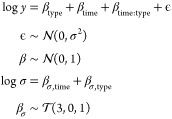
19Model 2
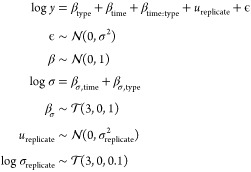
20Model 3
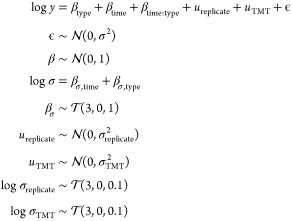
21Each of the models progresses with more complexity. We compute the posterior model probabilities for each of these examples and find that , , and  (see the vignette in the Supporting Information for more details). This suggest that a group-level effect for replicates is warranted, but there is less support for the more complex model 3. Note that because computing the posterior model probabilities includes integration against the prior, these probabilities are automatically penalized for model complexity. See the accompanying vignette in the Supporting Information for further exploration.

#### 2.3.7 Using Uncertainty from a Bayesian Analysis

Bayesians quantify uncertainty using probability distributions. Perhaps the most commonly used representation of uncertainty is the credible interval.^35^ A credible interval is an interval (*a*, *b*) such that a parameter lies within this interval with some probability. For example, we could ask for an interval such that the probability that a protein’s log abundance falls between *a* and *b* with probability 0.95. In notation used earlier *P*(*a* <  log *x* < *b*) = 0.95. We can see that the interval (*a*, *b*) is not unique.

The analogous quantity in frequentist statistics is the confidence interval; however, it is an entirely different concept. This is seen most clearly by asking which parts of the constructions are random. For credible intervals, it is the quantity of interest θ that is random and the interval that is a fixed quantity, while for a confidence interval the parameter is fixed, and the interval is random, since it depends on the randomly observed sample.

However, Bayesians can report any quantity that can be derived from the posterior distribution or posterior predictive distribution, which in practice can be a very complex representation of uncertainty. Since summarization can distort the representation of uncertainty, we recommend reporting the full posterior distribution whenever that is practical.

**Figure 5:**
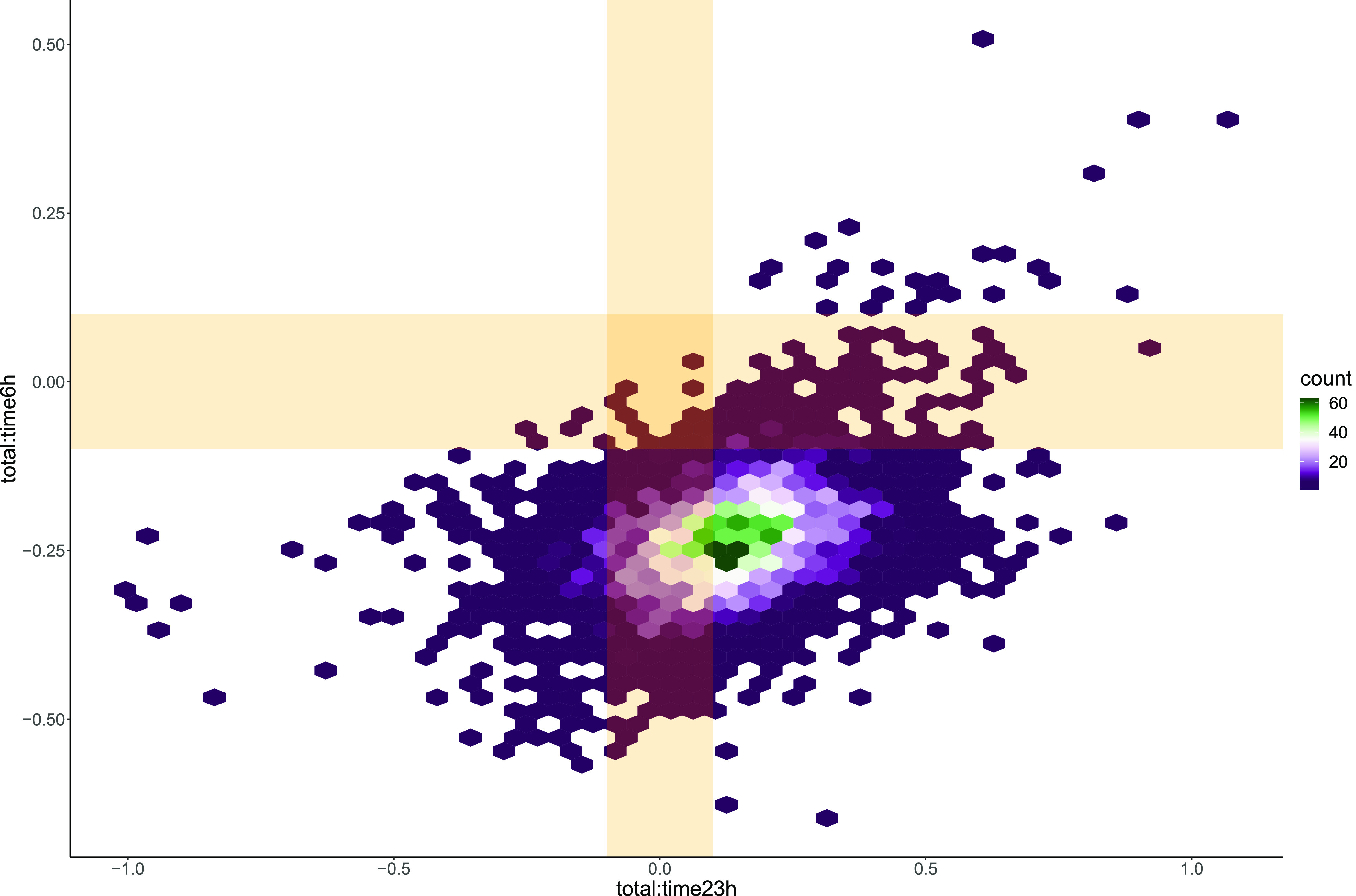
Joint posterior distribution of interaction effects. Joint posterior distribution of the interaction effect of type with time at 6 h and 23 h. The distribution is shown as a 2D histogram with a hexagon-based density estimation. Orange regions highlight absolute effect sizes less than 0.1. We observe that the density is concentrated outside this region, though it is more overlapped at 23 h.

For our OOPS example, we are interested in the interaction effects, since these allow us to determine whether the proportion of protein bound to RNA is changing between conditions. In Figure 5, we plot the joint distribution of the two interaction effects. We can then ask a number of question of this joint distribution. Some examples include the probability of being positive or negative, the probability of having the opposite signs, the probability that the absolute effects exceed 0.1, and many more (see the accompanying vignette in the Supporting Information). One possible deduction from Figure 5 is that the probability that the interaction effect changes sign from negative to positive is 0.779. This means that it is likely that the proportion of protein bound to RNA is depleting at 6 h while it is increasing at 23 h. This suggests that this RNA-binding protein is functionally relevant during the cell cycle.

### 2.4 Modeling Strategies

#### 2.4.1 Parametric Models

Here, we outline some commonly used modeling strategies and relate them to the proteomics literature. This is not meant to be exhaustive, nor could it be, since there are infinitely many possible models one could specify. One of the most commonly used models is the linear model, where we wish to link a set of predictors to outcomes:
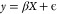
22

If we choose ϵ to be Gaussian noise, we can describe the model as follows:
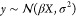
23

There is nothing Bayesian about this model until we specify priors. Remember, the choice of prior should be motivated by generative and predictive modeling, and of course the priors should respect the domain of the parameters. Typically, one may start with a Gaussian or Student-*t* prior on β. The prior on σ could be specified from a variety of probability distributions that respect positivity. Recommendations usually include half-normal, exponential, half-Student-*t*, and half-Cauchy depending on how confident we are about the scale of the noise.^36^ Since protein abundances are positive quantities, it is typical to model them as a log-normal distribution:
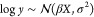
24

If our observed data were counts, then it may be sensible to use Poisson or negative binomial regression:^53^
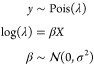
25and
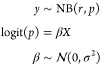
26

In each of the above cases, we would have to choose appropriate priors on the model parameters (again, using the evaluation strategies previously discussed). Many data have an excess of zeros which are not captured by the usual statistical models. Many distributions can be extended to a hurdle model or a zero-inflated model to account for these observations. The distribution of the noise process can be, again, as exotic as needed for the task at hand. Consistent outliers might call for a Student-*t* distribution, or perhaps the noise itself depends on some covariates, such as time or spatial location. See Goeminne et al.^40^ for an example, albeit non-Bayesian, of a hurdle model applied to proteomics.

Another useful model strategy is to allow parameters at the population level and group level; note that these are sometimes referred to as fixed and random effects. For example, a paired *t* test is a linear model with grouping specified by the subject or replicate. More complex groupings are allowed, including interactions between groupings and groups that are nested within each other. If β and *u* are population-level and group-level coefficients with design matrices *X* and *Z*, then a log linear (mixed) model would be^4^
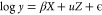
27

Group-level parameters (random/mixed effects models) allow us to model the nested and hierarchical variability that is associated with typical proteomics experiments. For example, a proteomics experiment may have biological and technical/injection replicates. The number of replicates performed is arbitrary, and thus, a replicate is a sample (in the probabilistic sense) from the distribution of replicates. We can then model replicates as if they add noise to a true biological signal β. Let ν_*j*_ be the noise added by technical replicate *j* and η_*k*_ be the noise added by biological replicate *k*. The log abundance of protein *i* in technical replicate *j* in biological replicate *k* could then be modeled as
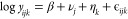
28

As before, the flexibility of the Bayesian analysis allows you to build any sensible probability distribution on top of this initial model. See Morris et al.^70,71^ for an application of mixed-models to proteomics data, as well as the accompanying vignette in the Supporting Information.

Another useful modeling strategy is mixture models, which occur frequently in the context of clustering and classification.^67^ The mixture model assumes that data arise from different components each with the same parametric density with different parameters:
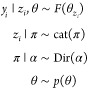
29

The priors and the likelihood can be chosen based on the specific application at hand, and the workflow recommendations can be applied. It is often insightful to write, using the law of total probability, the mixture model as
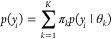
30

Note that because a Dirichlet prior is placed on π, the entries must all be non-negative and sum to unity. Hence, the entries of π can be interpreted as weights. The data cluster by being associated to the component density which fits those observations through the variables *z*_*i*_. Examples of mixture models applied to proteomics include Chung et al.^18^ and Crook et al.^20,21^

#### 2.4.2 Nonparametric Models

In contrast to parametric models, nonparametric models allow more parameters as more data are observed. Phrased another way, in a parametric model, there are finitely many parameters, while in a nonparametric model, there are infinitely many such parameters. This makes nonparametric models more flexible; however, to avoid the overfitting concerns raised in earlier sections, we ought to be prudent with our choice of priors. One of the most popular nonparametric models is the Gaussian process (GP), which can be used to model functions *f*. Suppose we observe data {(*x*_*i*_, *y*_*i*_)_*i*=1,···,*n*_}; we wish to find a function *f* such that *f*(*x*_*i*_) models *y*_*i*_. Let us assume a Gaussian regression setup, using a *Gaussian process prior* to model *f*:
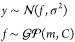
31

The Gaussian process is a distribution over *functions* that is uniquely characterized by its mean and covariance functions. The choices of mean and covariance functions are modeling choices to be made by the domain expert. Typically, the covariance function is parametrized by some parameters *C* = *C*(θ), and we can also place priors on these parameters so that θ ∼ *p*(θ). Again, these modeling choices can be evaluated using prior/posterior predictive checks. We refer to several discussion on choosing priors for Gaussian processes.^5,77,25,102,30^ For applications of the Gaussian process to proteomics data, see Maboudi Afkham et al.,^60^ Crook et al.,^21^ Shin et al.,^92^ and Fang et al.^28^

The other nonparametric model that is frequently used is the Dirichlet process.^29,1^ Dirichlet processes are a popular tool for modeling data with parameter repetitions. For example, when we cluster data, all observation associated with cluster 1 share the same parameter θ_1_. The Dirichlet process is defined using a base distribution *G* and a concentration parameter α and is written DP(*G*, α). For example, suppose that ; then, we can simulate from the Dirichlet process as follows. For any *i* ≥ 1, with probability , sample , and with probability , let *x*_*i*_ = *x*, where *n*_*x*_ is the number of previous observations of *x*. This means that if we have already observed a value, then we are increasingly likely to observe it in the future. This property is sometime referred to as the “rich get richer property”.

The Dirichlet process allows us to work with mixture models with infinitely many components, which is useful for characterizing the uncertainty in the number of components. Once we have a sensible parametric likelihood for the observations *F*(θ_*i*_), the Dirichlet process can be used as a prior to construct the Dirichlet process mixture model:
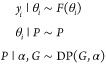
32

Since *P* will be discrete, the set{θ_*i*_}_*i*=1,···,*n*_ will contain repetitions. This allows us to think of this model as a mixture model, where the groups of parameters define the components. Extensions are available,^96,83^ and MCMC algorithms for fitting these models can be found in a work by Neal.^72^ For applications of Dirichlet processes to proteomics data, see Claassen et al.^19^ and Choi et al.^16^

## 3 Discussion

Despite Bayesian statistics offering a powerful and flexible framework for performing a proteomics data analysis, only a few problems have yet been tackled using this methodology. Even when Bayesian statistics has been applied, the methodology has not made complete use of the information available from such an analysis. Many analyses have simply resorted to proxies from frequentist-based approaches. One of the key advantages of the Bayesian approach is to be able to jointly model several quantities and provide uncertainty estimates in any parameters. Another advantage of Bayesian statistics is that it makes modeling assumptions explicit; hence, it becomes clear how the models can be improved and what is the extent of their limitations.

Here, we have summarized key modeling ideas in Bayesian statistics starting with the workflow. We have highlighted that the Bayesian workflow has a consistent approach to model building, model criticism, and evaluation grounded in probability theory. Using a case study, we have provided a workflow for developing a Bayesian model for organic orthogonal phase separation (OOPS) data. We then proceeded to describe and illustrate common modeling strategies to help proteomics researchers understand key models in the literature and link them to current methods used in the literature.

Mass-spectrometry-based proteomics appears to have resisted uptake on Bayesian methods for various reasons. These include, but are not limited to, lack of familiarity with the workflow and tools available, lack of compelling examples in the literature, and lack of desire to invest in bespoke model development. We hope that this Review goes some way in removing some of these barriers to applying and understanding Bayesian methods.

## Supporting Information Available

Additional figures including a heatmap of amino-acid probabilities and spectra and contour spectra of PARRDAARA (PDF)
Additional files including Data S1 (oopsdata, for OOPS modeling); Material S1 (oops_modelling, vignette for data exploration and straightforward modeling); and Material S2 (oops_modelling_part2, vignette for advanced modeling) (ZIP)

## Notes

*Data Availability*. Data to reproduce the figures are provided in the Supporting Information. Experimental data are available from the original manuscripts.

## Notes

*Code Availability*. Code is available from the manuscript github repository https://github.com/ococrook/2021-BayesProtReview.
