# Supplementary material for: Challenges and Opportunities for Bayesian Statistics in Proteomics
Source: J Proteome Res. 2022 Mar 8;21(4):849–64. doi: 10.1021/acs.jproteome.1c00859 (PMC8982455; doi:10.1021/acs.jproteome.1c00859)
Supplement: Supplementary file 1 — pr1c00859_si_001.pdf [file pr1c00859_si_001.pdf]

Supplement: Challenges and opportunities for Bayesian statistics in proteomics

Charlotte M. Deane

February 9, 2022

1 Contents

1. Figure S1: Heatmap of amino-acid probabilities
2. Figure S2: Spectra of PARRDAARA
3. FIgure S3: Contour Spectra of PARRDAARA
4. Data S1: oopsdata, Data for oops modelling (separate file).
5. Material S1: oops_modelling, supplementary vignette for data exploration and straightforward modelling. (separate file)
6. Material S2: oops_modelling_part2, supplementary vignette for advanced modelling. (separate file)

2 Representing uncertainty in common mass-spectrometry quantities

One quantity that we frequently interested in mass-spectrometry is the sequence of a peptide. There is some uncertainty in a peptide sequence given the relevant spectra. If we quantified the probability in this sequence, we could plot the probability that one amino acid proceeds another as a heatmap, example below:

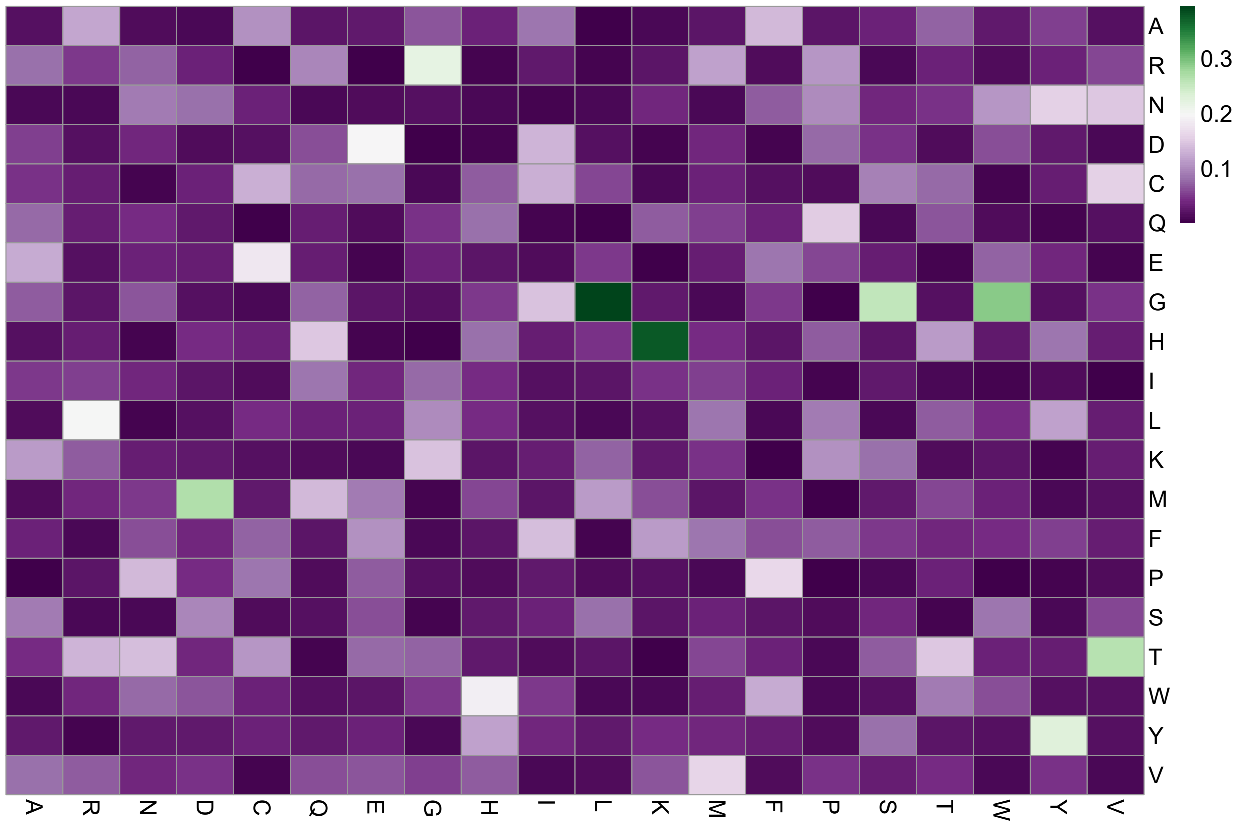

Figure S1: **Heatmap of amino-acid probabilities.** A heatmap where the the $(i,j)^{th}$ entry represents the probability that amino acid i is followed by amino acid j

.

Another quantity is the uncertainty in a spectra. The following are 9 MS1 spectra which are all compatible with peptide PARRDAARA with total intensity 1000.

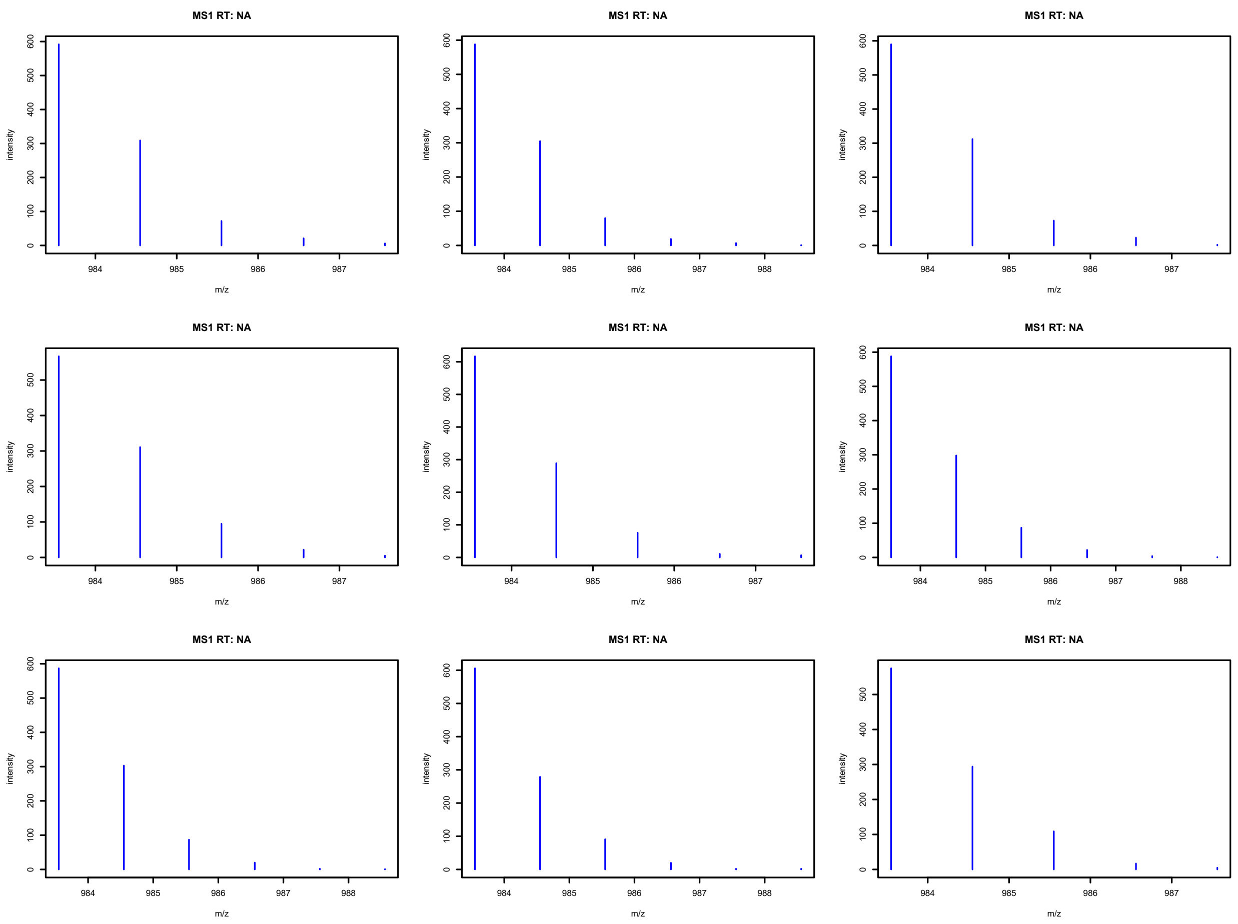

Figure S2: **Spectra of PARRDAARA.** Spectra diagrams showing spectra compatible with the MS1 spectra of PARRDAARA

.

It is infeasible to plot the 1000's of spectra that are compatible with MS1 spectra of an amino-acid sequence. Hence, we can use a contour plot which capture the local density of the spectra locations and heights. A typical spectra is overlaid for reference in white.

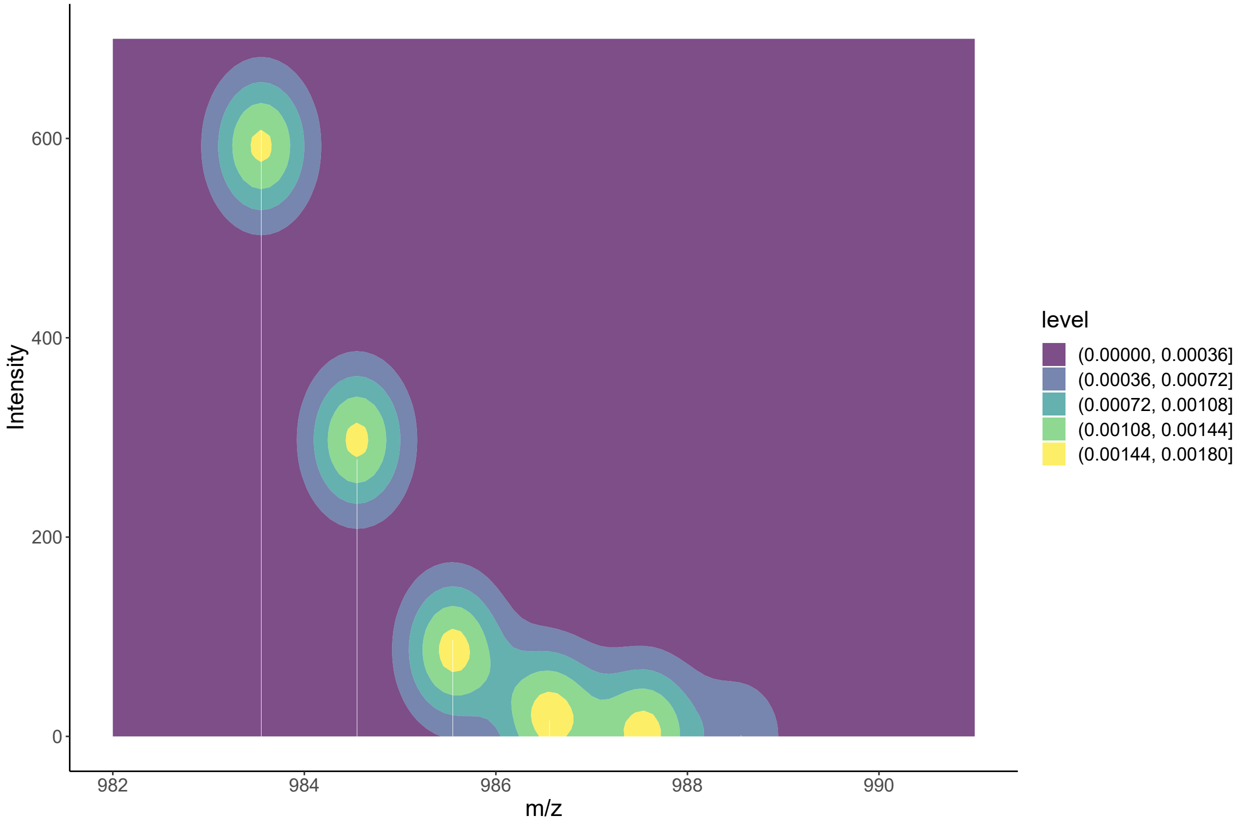

Figure S3: **Contour Spectra of PARRDAARA.** A contoured Spectra diagram showing the densities compatible with the MS1 spectra of PARRDAARA. Colour bar indicated relative probability. Example spectra overlaid in white.
