# Supplementary material for: Challenges and Opportunities for Bayesian Statistics in Proteomics
Source: J Proteome Res. 2022 Mar 8;21(4):849–64. doi: 10.1021/acs.jproteome.1c00859 (PMC8982455; doi:10.1021/acs.jproteome.1c00859)
Supplement: Supplementary file 2 — pr1c00859_si_002.zip [file pr1c00859_si_002.zip › Supporting information for publication/oops_modelling.html]

Bayesian analysis of Organic Orthogonal Phase Seperation Data


# Bayesian analysis of Organic Orthogonal Phase Seperation Data

Oliver M. Crook

#### 22/09/2021

# 1 Introduction

Here, we attempt to build a principled Bayesian model for some OOPS data.
We illustrate the main ideas of a Bayesian workflow and refer to the
accompanying manuscript for relevent details. The first code chunk loads the
packages we are interested in.

```
library(brms)
```

```
## Warning: package 'brms' was built under R version 4.0.5
```

```
## Loading required package: Rcpp
```

```
## Loading 'brms' package (version 2.15.0). Useful instructions
## can be found by typing help('brms'). A more detailed introduction
## to the package is available through vignette('brms_overview').
```

```
## 
## Attaching package: 'brms'
```

```
## The following object is masked from 'package:stats':
## 
##     ar
```

```
library(pheatmap)
library(RColorBrewer)
library(MSnbase)
```

```
## Loading required package: BiocGenerics
```

```
## Warning: package 'BiocGenerics' was built under R version 4.0.5
```

```
## Loading required package: parallel
```

```
## 
## Attaching package: 'BiocGenerics'
```

```
## The following objects are masked from 'package:parallel':
## 
##     clusterApply, clusterApplyLB, clusterCall, clusterEvalQ,
##     clusterExport, clusterMap, parApply, parCapply, parLapply,
##     parLapplyLB, parRapply, parSapply, parSapplyLB
```

```
## The following objects are masked from 'package:stats':
## 
##     IQR, mad, sd, var, xtabs
```

```
## The following objects are masked from 'package:base':
## 
##     anyDuplicated, append, as.data.frame, basename, cbind, colnames,
##     dirname, do.call, duplicated, eval, evalq, Filter, Find, get, grep,
##     grepl, intersect, is.unsorted, lapply, Map, mapply, match, mget,
##     order, paste, pmax, pmax.int, pmin, pmin.int, Position, rank,
##     rbind, Reduce, rownames, sapply, setdiff, sort, table, tapply,
##     union, unique, unsplit, which.max, which.min
```

```
## Loading required package: Biobase
```

```
## Welcome to Bioconductor
## 
##     Vignettes contain introductory material; view with
##     'browseVignettes()'. To cite Bioconductor, see
##     'citation("Biobase")', and for packages 'citation("pkgname")'.
```

```
## Loading required package: mzR
```

```
## Warning in fun(libname, pkgname): mzR has been built against a different Rcpp version (1.0.5)
## than is installed on your system (1.0.6). This might lead to errors
## when loading mzR. If you encounter such issues, please send a report,
## including the output of sessionInfo() to the Bioc support forum at 
## https://support.bioconductor.org/. For details see also
## https://github.com/sneumann/mzR/wiki/mzR-Rcpp-compiler-linker-issue.
```

```
## Loading required package: S4Vectors
```

```
## Loading required package: stats4
```

```
## 
## Attaching package: 'S4Vectors'
```

```
## The following object is masked from 'package:base':
## 
##     expand.grid
```

```
## Loading required package: ProtGenerics
```

```
## 
## Attaching package: 'ProtGenerics'
```

```
## The following object is masked from 'package:stats':
## 
##     smooth
```

```
## 
## This is MSnbase version 2.16.1 
##   Visit https://lgatto.github.io/MSnbase/ to get started.
```

```
## 
## Attaching package: 'MSnbase'
```

```
## The following objects are masked from 'package:ProtGenerics':
## 
##     executeProcessingStep, ProcessingStep
```

```
## The following object is masked from 'package:base':
## 
##     trimws
```

```
library(ggplot2)
```

```
## Warning: package 'ggplot2' was built under R version 4.0.5
```

```
library(ggfortify)
bayesplot::color_scheme_set("teal")
```

Now, we load the data we are interested in, which is from Queiroz et al. 2019.
The data have already been summarised to protein level and column median normalised.
The design used 2 seperate TMT 10-plexes to measure total protein abundance and
oops samples. This allows us to model changes in RNA binding that are independent
of concurrent changes in total protien abundance. Changes in oops-enriched
protein abundance samples that do not correlated with total protein
abundance changes are indicative of specific RBPs binding RNA differentially
in those conditions. The experimental design uses thymidine-nocodazole arrest
and then measures protein abundance, as well as oops enriched protein
abundance at \(0,6\) and \(23\) hours. Triplicate measurements are made, except for
at 6 hours where 4 replicates where taken.
For further details see Queiroz et al. 2019

```
# We assume the data is in the working directory
oopsdata <- readMSnSet2(file = "oopsdata.csv", ecol = 2:21, fnames = 1)
```

First, it is a good idea to get a feel for the data using some exploratory data
analysis. Below we produce a heatmap. We can easily see from this data that there
are some differences between the total and oops samples, as well as differences
between the timepoints.

```
pheatmap(exprs(oopsdata), col = brewer.pal(n = 10, name = "PRGn"), cluster_cols = FALSE)
```

Now we perform PCA to see if there is any systematic clustering. There
are clear differences between the oops and total samples, as well
as a difference between 6h oops and 23h oops samples.

```
autoplot(prcomp(t(exprs(oopsdata))), label = TRUE) + theme_classic()
```

Now, we look at a global boxplot across the columns. There looks to fairly
consistent distributions of intensities across all the columns and perhaps
slightly higher variance for the oops samples. We visualise
the varainces in a boxplot

```
boxplot(exprs(oopsdata), col = rep(brewer.pal(n = 2, name = "Set2"), each = 10))
```

```
## Warning in brewer.pal(n = 2, name = "Set2"): minimal value for n is 3, returning requested palette with 3 different levels
```

```
barplot(apply(exprs(oopsdata),2,var), col = rep(brewer.pal(n = 2, name = "Set2"), each = 10))
```

```
## Warning in brewer.pal(n = 2, name = "Set2"): minimal value for n is 3, returning requested palette with 3 different levels
```

# 2 Initial modelling thoughts

The first step is to establish what questions and aspects of the experimental
design we are interested in modelling. Ultimately, we are interested in looking
at RBPs binding RNA differentially. However, simply a change in abundance
will make it appear like there is more RNA binding. We need to control
simultaniously for the changes in abundance. There are also changes across
the different times points. We also make replicate measurements and should
allow some modelled variation for replicates. There are also TMT tags, we might
expect some variation due to which TMT tag was used. The variance is also
higher for the oops sample so it may be sensible to allow the variance to depend
on the sample type.

We first buid the simplest model that answers the question we are interested in.
We account for time and sample type (oops vs total) and the interaction between them.
The interaction allows us to examine RBPs that bind RNA differentially. The model
looks like following for each protein, with Gaussian noice
\[
\log abundance = total + time + total\*time
\]
We test using a single protein for simplicity, the code chunk below extracts
this protein.

```
set.seed(1)
data <- data.frame(exprs(oopsdata)[sample.int(nrow(oopsdata), size = 1),])
colnames(data) <- "abundance"
data$type <- as.factor(rep(c("total", "oops"), each = 10))
data$time <- as.factor(rep(rep(c("0", "6", "23"), times = c(3,4,3)), 2))
data$replicate <- as.factor(sapply(strsplit(rownames(data), "_"), function(x)x[2]))
data$tag <- as.factor(rep(TMT10@reporterNames, times = 2))
```

# 3 Fitting our first model

Let us start the workflow by setting some priors and the model. In the code
chunk below, we pick some roughly arbitrary priors to get started. When
developing your own model it can be useful to use the `get_prior` function to
know which priors to set.

Below, we have asked the code just to return samples from the prior so we can see
what effect they have in practice. We can then visualise some summaries of the
data.

```
pr <- c(prior(normal(0,1), class = "b"),
  prior(exponential(1), class = "sigma"))


fit_prot1 <- brm(abundance ~ 0 + type + time + (type:time),
                 data = data,
                 family = gaussian(link = "identity"),
                 sample_prior = "only",
                 prior = pr)
```

```
## Compiling Stan program...
```

```
## Start sampling
```

```
## 
## SAMPLING FOR MODEL '95b1aa6f5505af21af888d28577bf886' NOW (CHAIN 1).
## Chain 1: 
## Chain 1: Gradient evaluation took 0 seconds
## Chain 1: 1000 transitions using 10 leapfrog steps per transition would take 0 seconds.
## Chain 1: Adjust your expectations accordingly!
## Chain 1: 
## Chain 1: 
## Chain 1: Iteration:    1 / 2000 [  0%]  (Warmup)
## Chain 1: Iteration:  200 / 2000 [ 10%]  (Warmup)
## Chain 1: Iteration:  400 / 2000 [ 20%]  (Warmup)
## Chain 1: Iteration:  600 / 2000 [ 30%]  (Warmup)
## Chain 1: Iteration:  800 / 2000 [ 40%]  (Warmup)
## Chain 1: Iteration: 1000 / 2000 [ 50%]  (Warmup)
## Chain 1: Iteration: 1001 / 2000 [ 50%]  (Sampling)
## Chain 1: Iteration: 1200 / 2000 [ 60%]  (Sampling)
## Chain 1: Iteration: 1400 / 2000 [ 70%]  (Sampling)
## Chain 1: Iteration: 1600 / 2000 [ 80%]  (Sampling)
## Chain 1: Iteration: 1800 / 2000 [ 90%]  (Sampling)
## Chain 1: Iteration: 2000 / 2000 [100%]  (Sampling)
## Chain 1: 
## Chain 1:  Elapsed Time: 0.026 seconds (Warm-up)
## Chain 1:                0.032 seconds (Sampling)
## Chain 1:                0.058 seconds (Total)
## Chain 1: 
## 
## SAMPLING FOR MODEL '95b1aa6f5505af21af888d28577bf886' NOW (CHAIN 2).
## Chain 2: 
## Chain 2: Gradient evaluation took 0 seconds
## Chain 2: 1000 transitions using 10 leapfrog steps per transition would take 0 seconds.
## Chain 2: Adjust your expectations accordingly!
## Chain 2: 
## Chain 2: 
## Chain 2: Iteration:    1 / 2000 [  0%]  (Warmup)
## Chain 2: Iteration:  200 / 2000 [ 10%]  (Warmup)
## Chain 2: Iteration:  400 / 2000 [ 20%]  (Warmup)
## Chain 2: Iteration:  600 / 2000 [ 30%]  (Warmup)
## Chain 2: Iteration:  800 / 2000 [ 40%]  (Warmup)
## Chain 2: Iteration: 1000 / 2000 [ 50%]  (Warmup)
## Chain 2: Iteration: 1001 / 2000 [ 50%]  (Sampling)
## Chain 2: Iteration: 1200 / 2000 [ 60%]  (Sampling)
## Chain 2: Iteration: 1400 / 2000 [ 70%]  (Sampling)
## Chain 2: Iteration: 1600 / 2000 [ 80%]  (Sampling)
## Chain 2: Iteration: 1800 / 2000 [ 90%]  (Sampling)
## Chain 2: Iteration: 2000 / 2000 [100%]  (Sampling)
## Chain 2: 
## Chain 2:  Elapsed Time: 0.032 seconds (Warm-up)
## Chain 2:                0.028 seconds (Sampling)
## Chain 2:                0.06 seconds (Total)
## Chain 2: 
## 
## SAMPLING FOR MODEL '95b1aa6f5505af21af888d28577bf886' NOW (CHAIN 3).
## Chain 3: 
## Chain 3: Gradient evaluation took 0 seconds
## Chain 3: 1000 transitions using 10 leapfrog steps per transition would take 0 seconds.
## Chain 3: Adjust your expectations accordingly!
## Chain 3: 
## Chain 3: 
## Chain 3: Iteration:    1 / 2000 [  0%]  (Warmup)
## Chain 3: Iteration:  200 / 2000 [ 10%]  (Warmup)
## Chain 3: Iteration:  400 / 2000 [ 20%]  (Warmup)
## Chain 3: Iteration:  600 / 2000 [ 30%]  (Warmup)
## Chain 3: Iteration:  800 / 2000 [ 40%]  (Warmup)
## Chain 3: Iteration: 1000 / 2000 [ 50%]  (Warmup)
## Chain 3: Iteration: 1001 / 2000 [ 50%]  (Sampling)
## Chain 3: Iteration: 1200 / 2000 [ 60%]  (Sampling)
## Chain 3: Iteration: 1400 / 2000 [ 70%]  (Sampling)
## Chain 3: Iteration: 1600 / 2000 [ 80%]  (Sampling)
## Chain 3: Iteration: 1800 / 2000 [ 90%]  (Sampling)
## Chain 3: Iteration: 2000 / 2000 [100%]  (Sampling)
## Chain 3: 
## Chain 3:  Elapsed Time: 0.024 seconds (Warm-up)
## Chain 3:                0.024 seconds (Sampling)
## Chain 3:                0.048 seconds (Total)
## Chain 3: 
## 
## SAMPLING FOR MODEL '95b1aa6f5505af21af888d28577bf886' NOW (CHAIN 4).
## Chain 4: 
## Chain 4: Gradient evaluation took 0 seconds
## Chain 4: 1000 transitions using 10 leapfrog steps per transition would take 0 seconds.
## Chain 4: Adjust your expectations accordingly!
## Chain 4: 
## Chain 4: 
## Chain 4: Iteration:    1 / 2000 [  0%]  (Warmup)
## Chain 4: Iteration:  200 / 2000 [ 10%]  (Warmup)
## Chain 4: Iteration:  400 / 2000 [ 20%]  (Warmup)
## Chain 4: Iteration:  600 / 2000 [ 30%]  (Warmup)
## Chain 4: Iteration:  800 / 2000 [ 40%]  (Warmup)
## Chain 4: Iteration: 1000 / 2000 [ 50%]  (Warmup)
## Chain 4: Iteration: 1001 / 2000 [ 50%]  (Sampling)
## Chain 4: Iteration: 1200 / 2000 [ 60%]  (Sampling)
## Chain 4: Iteration: 1400 / 2000 [ 70%]  (Sampling)
## Chain 4: Iteration: 1600 / 2000 [ 80%]  (Sampling)
## Chain 4: Iteration: 1800 / 2000 [ 90%]  (Sampling)
## Chain 4: Iteration: 2000 / 2000 [100%]  (Sampling)
## Chain 4: 
## Chain 4:  Elapsed Time: 0.028 seconds (Warm-up)
## Chain 4:                0.025 seconds (Sampling)
## Chain 4:                0.053 seconds (Total)
## Chain 4:
```

```
plot(fit_prot1, ask = FALSE)
```

```
summary(fit_prot1)
```

```
##  Family: gaussian 
##   Links: mu = identity; sigma = identity 
## Formula: abundance ~ 0 + type + time + (type:time) 
##    Data: data (Number of observations: 20) 
## Samples: 4 chains, each with iter = 2000; warmup = 1000; thin = 1;
##          total post-warmup samples = 4000
## 
## Population-Level Effects: 
##                  Estimate Est.Error l-95% CI u-95% CI Rhat Bulk_ESS Tail_ESS
## typeoops             0.00      1.00    -1.94     1.99 1.00     6472     2922
## typetotal            0.01      0.97    -1.87     1.89 1.00     6180     3166
## time23              -0.02      1.02    -2.03     2.03 1.00     6985     2957
## time6                0.02      0.97    -1.87     1.95 1.00     7031     3117
## typetotal:time23     0.01      1.04    -2.00     2.08 1.00     6071     2729
## typetotal:time6      0.01      1.00    -1.96     1.92 1.00     5986     2945
## 
## Family Specific Parameters: 
##       Estimate Est.Error l-95% CI u-95% CI Rhat Bulk_ESS Tail_ESS
## sigma     1.00      1.01     0.02     3.65 1.00     3703     1801
## 
## Samples were drawn using sampling(NUTS). For each parameter, Bulk_ESS
## and Tail_ESS are effective sample size measures, and Rhat is the potential
## scale reduction factor on split chains (at convergence, Rhat = 1).
```

We make a few initial observation. For the moment is appears that the parameters
are on a sensible scale. There are a few aspects we should be mindful of as we
progress through the modelling. The first is the values for \(\sigma\), which can
occasionally get unrealistically large. For these data standard deviations above
\(2.5\) should be extremely rare, whist we see from the prior simulations that this
can happen not infrequently. We also note, from examining the PCA plot, that
the effect size for type (oops or total) is likely to be larger than that for
time. Furthermore, it seems unlikely that we expect interaction effects to be
on the same scale as type changes. One might expect that after account for
the type and time that the interaction would have a smaller effect.

# 4 Prior Predictive check

The next step in the modelling is a prior predictive check. Here, we produce
some summary of the data and summaries of the data simulated from the prior
predictive distribution. A huge number of different checks are available.
Visually the simulated samples ought to “look like” the observed data.

```
# compute kernel density estimate of data and simulated data and plot
pp_check(object = fit_prot1, "dens_overlay")
```

```
## Using 10 posterior samples for ppc type 'dens_overlay' by default.
```

```
# plot data in a histogram to see distributions
pp_check(object = fit_prot1, "hist")
```

```
## Using 10 posterior samples for ppc type 'hist' by default.
```

```
## `stat_bin()` using `bins = 30`. Pick better value with `binwidth`.
```

```
# plot data in a boxplot to see distribtuions
pp_check(object = fit_prot1, "boxplot")
```

```
## Using 10 posterior samples for ppc type 'boxplot' by default.
```

```
## notch went outside hinges. Try setting notch=FALSE.
## notch went outside hinges. Try setting notch=FALSE.
## notch went outside hinges. Try setting notch=FALSE.
## notch went outside hinges. Try setting notch=FALSE.
```

```
# produce summary statistics
pp_check(object = fit_prot1, "stat_2d")
```

```
## Using all posterior samples for ppc type 'stat_2d' by default.
```

```
# produce intervals
pp_check(object = fit_prot1, "intervals")
```

```
## Using all posterior samples for ppc type 'intervals' by default.
```

```
# predctive errors
pp_check(object = fit_prot1, "error_hist")
```

```
## Using 10 posterior samples for ppc type 'error_hist' by default.
```

```
## `stat_bin()` using `bins = 30`. Pick better value with `binwidth`.
```

These prior predictive checks suggest that the prior is centered correctly,
but is very diffuse. In principle, a diffuse prior might not be a problem if
the data are sufficiently informative. However, for proteomics data we are unlikely
to make more than 3 or 4 measurements and it maybe sensible to build a better
generative model before we move further into the modelling task.

Let’s us be a little more mathematical than our previous approach. The standard
deviations are unlikely to exceed \(2.5\) in this proteomics experiments. However,
an `exponential(1)` prior leads to standard deviations above 2.5 around 9% of the
time (see code chunk below). For us rare would be 1 in 5000 proteins so this
is clearly miscalibrated with our expectations. We can see that an exponential
with rate of \(4\) capture this expectation.

```
# probability of observing sd above 2.5
sum(rexp(1000, rate = 1) > 2.5)/1000
```

```
## [1] 0.07
```

```
# with rate 4, probability of observing a sd above 2.5 is 1/5000
sum(rexp(10000, rate = 4) > 2.5)/10000
```

```
## [1] 1e-04
```

However, as we push the prior through the model is not clear how changing
the prior on the standard deviation will be reflected in generated data. We
can repeat our prior predictive checks. Below we fit the model without data
as before. Note that values for \(\sigma\) are now more aligned with our expectations.

```
pr <- c(prior(normal(0,1), class = "b"),
  prior(exponential(4), class = "sigma"))


fit_prot1 <- brm(abundance ~ 0 + type + time + (type:time),
                 data = data,
                 family = gaussian(link = "identity"),
                 sample_prior = "only",
                 prior = pr)
```

```
## Compiling Stan program...
```

```
## Start sampling
```

```
## 
## SAMPLING FOR MODEL 'f3ea8e7e93c0ee0812911bccee541a3c' NOW (CHAIN 1).
## Chain 1: 
## Chain 1: Gradient evaluation took 0 seconds
## Chain 1: 1000 transitions using 10 leapfrog steps per transition would take 0 seconds.
## Chain 1: Adjust your expectations accordingly!
## Chain 1: 
## Chain 1: 
## Chain 1: Iteration:    1 / 2000 [  0%]  (Warmup)
## Chain 1: Iteration:  200 / 2000 [ 10%]  (Warmup)
## Chain 1: Iteration:  400 / 2000 [ 20%]  (Warmup)
## Chain 1: Iteration:  600 / 2000 [ 30%]  (Warmup)
## Chain 1: Iteration:  800 / 2000 [ 40%]  (Warmup)
## Chain 1: Iteration: 1000 / 2000 [ 50%]  (Warmup)
## Chain 1: Iteration: 1001 / 2000 [ 50%]  (Sampling)
## Chain 1: Iteration: 1200 / 2000 [ 60%]  (Sampling)
## Chain 1: Iteration: 1400 / 2000 [ 70%]  (Sampling)
## Chain 1: Iteration: 1600 / 2000 [ 80%]  (Sampling)
## Chain 1: Iteration: 1800 / 2000 [ 90%]  (Sampling)
## Chain 1: Iteration: 2000 / 2000 [100%]  (Sampling)
## Chain 1: 
## Chain 1:  Elapsed Time: 0.031 seconds (Warm-up)
## Chain 1:                0.031 seconds (Sampling)
## Chain 1:                0.062 seconds (Total)
## Chain 1: 
## 
## SAMPLING FOR MODEL 'f3ea8e7e93c0ee0812911bccee541a3c' NOW (CHAIN 2).
## Chain 2: 
## Chain 2: Gradient evaluation took 0 seconds
## Chain 2: 1000 transitions using 10 leapfrog steps per transition would take 0 seconds.
## Chain 2: Adjust your expectations accordingly!
## Chain 2: 
## Chain 2: 
## Chain 2: Iteration:    1 / 2000 [  0%]  (Warmup)
## Chain 2: Iteration:  200 / 2000 [ 10%]  (Warmup)
## Chain 2: Iteration:  400 / 2000 [ 20%]  (Warmup)
## Chain 2: Iteration:  600 / 2000 [ 30%]  (Warmup)
## Chain 2: Iteration:  800 / 2000 [ 40%]  (Warmup)
## Chain 2: Iteration: 1000 / 2000 [ 50%]  (Warmup)
## Chain 2: Iteration: 1001 / 2000 [ 50%]  (Sampling)
## Chain 2: Iteration: 1200 / 2000 [ 60%]  (Sampling)
## Chain 2: Iteration: 1400 / 2000 [ 70%]  (Sampling)
## Chain 2: Iteration: 1600 / 2000 [ 80%]  (Sampling)
## Chain 2: Iteration: 1800 / 2000 [ 90%]  (Sampling)
## Chain 2: Iteration: 2000 / 2000 [100%]  (Sampling)
## Chain 2: 
## Chain 2:  Elapsed Time: 0.028 seconds (Warm-up)
## Chain 2:                0.026 seconds (Sampling)
## Chain 2:                0.054 seconds (Total)
## Chain 2: 
## 
## SAMPLING FOR MODEL 'f3ea8e7e93c0ee0812911bccee541a3c' NOW (CHAIN 3).
## Chain 3: 
## Chain 3: Gradient evaluation took 0 seconds
## Chain 3: 1000 transitions using 10 leapfrog steps per transition would take 0 seconds.
## Chain 3: Adjust your expectations accordingly!
## Chain 3: 
## Chain 3: 
## Chain 3: Iteration:    1 / 2000 [  0%]  (Warmup)
## Chain 3: Iteration:  200 / 2000 [ 10%]  (Warmup)
## Chain 3: Iteration:  400 / 2000 [ 20%]  (Warmup)
## Chain 3: Iteration:  600 / 2000 [ 30%]  (Warmup)
## Chain 3: Iteration:  800 / 2000 [ 40%]  (Warmup)
## Chain 3: Iteration: 1000 / 2000 [ 50%]  (Warmup)
## Chain 3: Iteration: 1001 / 2000 [ 50%]  (Sampling)
## Chain 3: Iteration: 1200 / 2000 [ 60%]  (Sampling)
## Chain 3: Iteration: 1400 / 2000 [ 70%]  (Sampling)
## Chain 3: Iteration: 1600 / 2000 [ 80%]  (Sampling)
## Chain 3: Iteration: 1800 / 2000 [ 90%]  (Sampling)
## Chain 3: Iteration: 2000 / 2000 [100%]  (Sampling)
## Chain 3: 
## Chain 3:  Elapsed Time: 0.025 seconds (Warm-up)
## Chain 3:                0.028 seconds (Sampling)
## Chain 3:                0.053 seconds (Total)
## Chain 3: 
## 
## SAMPLING FOR MODEL 'f3ea8e7e93c0ee0812911bccee541a3c' NOW (CHAIN 4).
## Chain 4: 
## Chain 4: Gradient evaluation took 0 seconds
## Chain 4: 1000 transitions using 10 leapfrog steps per transition would take 0 seconds.
## Chain 4: Adjust your expectations accordingly!
## Chain 4: 
## Chain 4: 
## Chain 4: Iteration:    1 / 2000 [  0%]  (Warmup)
## Chain 4: Iteration:  200 / 2000 [ 10%]  (Warmup)
## Chain 4: Iteration:  400 / 2000 [ 20%]  (Warmup)
## Chain 4: Iteration:  600 / 2000 [ 30%]  (Warmup)
## Chain 4: Iteration:  800 / 2000 [ 40%]  (Warmup)
## Chain 4: Iteration: 1000 / 2000 [ 50%]  (Warmup)
## Chain 4: Iteration: 1001 / 2000 [ 50%]  (Sampling)
## Chain 4: Iteration: 1200 / 2000 [ 60%]  (Sampling)
## Chain 4: Iteration: 1400 / 2000 [ 70%]  (Sampling)
## Chain 4: Iteration: 1600 / 2000 [ 80%]  (Sampling)
## Chain 4: Iteration: 1800 / 2000 [ 90%]  (Sampling)
## Chain 4: Iteration: 2000 / 2000 [100%]  (Sampling)
## Chain 4: 
## Chain 4:  Elapsed Time: 0.027 seconds (Warm-up)
## Chain 4:                0.028 seconds (Sampling)
## Chain 4:                0.055 seconds (Total)
## Chain 4:
```

```
plot(fit_prot1, ask = FALSE)
```

```
summary(fit_prot1)
```

```
##  Family: gaussian 
##   Links: mu = identity; sigma = identity 
## Formula: abundance ~ 0 + type + time + (type:time) 
##    Data: data (Number of observations: 20) 
## Samples: 4 chains, each with iter = 2000; warmup = 1000; thin = 1;
##          total post-warmup samples = 4000
## 
## Population-Level Effects: 
##                  Estimate Est.Error l-95% CI u-95% CI Rhat Bulk_ESS Tail_ESS
## typeoops            -0.00      0.98    -1.90     1.97 1.00     6089     2964
## typetotal           -0.00      0.98    -1.93     1.90 1.00     7521     3236
## time23              -0.01      1.01    -1.98     2.02 1.00     6306     2979
## time6               -0.01      0.99    -1.94     1.92 1.00     6427     2254
## typetotal:time23     0.00      0.99    -1.95     1.87 1.00     7048     2922
## typetotal:time6      0.02      1.03    -2.04     2.08 1.00     6634     2837
## 
## Family Specific Parameters: 
##       Estimate Est.Error l-95% CI u-95% CI Rhat Bulk_ESS Tail_ESS
## sigma     0.25      0.25     0.01     0.90 1.00     5027     2192
## 
## Samples were drawn using sampling(NUTS). For each parameter, Bulk_ESS
## and Tail_ESS are effective sample size measures, and Rhat is the potential
## scale reduction factor on split chains (at convergence, Rhat = 1).
```

From the prior predictive checks below we see that the simulated data are much
less diffuse in the observed data than before, whilst still being fairly broad
in the range of data we could model.

```
# compute kernel density estimate of data and simulated data and plot
pp_check(object = fit_prot1, "dens_overlay")
```

```
## Using 10 posterior samples for ppc type 'dens_overlay' by default.
```

```
# plot data in a histogram to see distributions
pp_check(object = fit_prot1, "hist")
```

```
## Using 10 posterior samples for ppc type 'hist' by default.
```

```
## `stat_bin()` using `bins = 30`. Pick better value with `binwidth`.
```

```
# plot data in a boxplot to see distribtuions
pp_check(object = fit_prot1, "boxplot")
```

```
## Using 10 posterior samples for ppc type 'boxplot' by default.
```

```
## notch went outside hinges. Try setting notch=FALSE.
## notch went outside hinges. Try setting notch=FALSE.
## notch went outside hinges. Try setting notch=FALSE.
## notch went outside hinges. Try setting notch=FALSE.
## notch went outside hinges. Try setting notch=FALSE.
## notch went outside hinges. Try setting notch=FALSE.
```

```
# produce summary statistics
pp_check(object = fit_prot1, "stat_2d")
```

```
## Using all posterior samples for ppc type 'stat_2d' by default.
```

```
# produce intervals
pp_check(object = fit_prot1, "intervals")
```

```
## Using all posterior samples for ppc type 'intervals' by default.
```

```
# predctive errors
pp_check(object = fit_prot1, "error_hist")
```

```
## Using 10 posterior samples for ppc type 'error_hist' by default.
```

```
## `stat_bin()` using `bins = 30`. Pick better value with `binwidth`.
```

# 5 Fitting the model to the data

Let us now examine the model fitted to the data.

```
pr <- c(prior(normal(0,1), class = "b"),
  prior(exponential(4), class = "sigma"))


fit_prot1_post<- brm(abundance ~ 0 + type + time + (type:time),
                 data = data,
                 family = gaussian(link = "identity"),
                 sample_prior = "no",
                 prior = pr)
```

```
## Compiling Stan program...
```

```
## recompiling to avoid crashing R session
```

```
## Start sampling
```

```
## 
## SAMPLING FOR MODEL 'f3ea8e7e93c0ee0812911bccee541a3c' NOW (CHAIN 1).
## Chain 1: 
## Chain 1: Gradient evaluation took 0 seconds
## Chain 1: 1000 transitions using 10 leapfrog steps per transition would take 0 seconds.
## Chain 1: Adjust your expectations accordingly!
## Chain 1: 
## Chain 1: 
## Chain 1: Iteration:    1 / 2000 [  0%]  (Warmup)
## Chain 1: Iteration:  200 / 2000 [ 10%]  (Warmup)
## Chain 1: Iteration:  400 / 2000 [ 20%]  (Warmup)
## Chain 1: Iteration:  600 / 2000 [ 30%]  (Warmup)
## Chain 1: Iteration:  800 / 2000 [ 40%]  (Warmup)
## Chain 1: Iteration: 1000 / 2000 [ 50%]  (Warmup)
## Chain 1: Iteration: 1001 / 2000 [ 50%]  (Sampling)
## Chain 1: Iteration: 1200 / 2000 [ 60%]  (Sampling)
## Chain 1: Iteration: 1400 / 2000 [ 70%]  (Sampling)
## Chain 1: Iteration: 1600 / 2000 [ 80%]  (Sampling)
## Chain 1: Iteration: 1800 / 2000 [ 90%]  (Sampling)
## Chain 1: Iteration: 2000 / 2000 [100%]  (Sampling)
## Chain 1: 
## Chain 1:  Elapsed Time: 0.072 seconds (Warm-up)
## Chain 1:                0.075 seconds (Sampling)
## Chain 1:                0.147 seconds (Total)
## Chain 1: 
## 
## SAMPLING FOR MODEL 'f3ea8e7e93c0ee0812911bccee541a3c' NOW (CHAIN 2).
## Chain 2: 
## Chain 2: Gradient evaluation took 0 seconds
## Chain 2: 1000 transitions using 10 leapfrog steps per transition would take 0 seconds.
## Chain 2: Adjust your expectations accordingly!
## Chain 2: 
## Chain 2: 
## Chain 2: Iteration:    1 / 2000 [  0%]  (Warmup)
## Chain 2: Iteration:  200 / 2000 [ 10%]  (Warmup)
## Chain 2: Iteration:  400 / 2000 [ 20%]  (Warmup)
## Chain 2: Iteration:  600 / 2000 [ 30%]  (Warmup)
## Chain 2: Iteration:  800 / 2000 [ 40%]  (Warmup)
## Chain 2: Iteration: 1000 / 2000 [ 50%]  (Warmup)
## Chain 2: Iteration: 1001 / 2000 [ 50%]  (Sampling)
## Chain 2: Iteration: 1200 / 2000 [ 60%]  (Sampling)
## Chain 2: Iteration: 1400 / 2000 [ 70%]  (Sampling)
## Chain 2: Iteration: 1600 / 2000 [ 80%]  (Sampling)
## Chain 2: Iteration: 1800 / 2000 [ 90%]  (Sampling)
## Chain 2: Iteration: 2000 / 2000 [100%]  (Sampling)
## Chain 2: 
## Chain 2:  Elapsed Time: 0.059 seconds (Warm-up)
## Chain 2:                0.053 seconds (Sampling)
## Chain 2:                0.112 seconds (Total)
## Chain 2: 
## 
## SAMPLING FOR MODEL 'f3ea8e7e93c0ee0812911bccee541a3c' NOW (CHAIN 3).
## Chain 3: 
## Chain 3: Gradient evaluation took 0 seconds
## Chain 3: 1000 transitions using 10 leapfrog steps per transition would take 0 seconds.
## Chain 3: Adjust your expectations accordingly!
## Chain 3: 
## Chain 3: 
## Chain 3: Iteration:    1 / 2000 [  0%]  (Warmup)
## Chain 3: Iteration:  200 / 2000 [ 10%]  (Warmup)
## Chain 3: Iteration:  400 / 2000 [ 20%]  (Warmup)
## Chain 3: Iteration:  600 / 2000 [ 30%]  (Warmup)
## Chain 3: Iteration:  800 / 2000 [ 40%]  (Warmup)
## Chain 3: Iteration: 1000 / 2000 [ 50%]  (Warmup)
## Chain 3: Iteration: 1001 / 2000 [ 50%]  (Sampling)
## Chain 3: Iteration: 1200 / 2000 [ 60%]  (Sampling)
## Chain 3: Iteration: 1400 / 2000 [ 70%]  (Sampling)
## Chain 3: Iteration: 1600 / 2000 [ 80%]  (Sampling)
## Chain 3: Iteration: 1800 / 2000 [ 90%]  (Sampling)
## Chain 3: Iteration: 2000 / 2000 [100%]  (Sampling)
## Chain 3: 
## Chain 3:  Elapsed Time: 0.061 seconds (Warm-up)
## Chain 3:                0.064 seconds (Sampling)
## Chain 3:                0.125 seconds (Total)
## Chain 3: 
## 
## SAMPLING FOR MODEL 'f3ea8e7e93c0ee0812911bccee541a3c' NOW (CHAIN 4).
## Chain 4: 
## Chain 4: Gradient evaluation took 0 seconds
## Chain 4: 1000 transitions using 10 leapfrog steps per transition would take 0 seconds.
## Chain 4: Adjust your expectations accordingly!
## Chain 4: 
## Chain 4: 
## Chain 4: Iteration:    1 / 2000 [  0%]  (Warmup)
## Chain 4: Iteration:  200 / 2000 [ 10%]  (Warmup)
## Chain 4: Iteration:  400 / 2000 [ 20%]  (Warmup)
## Chain 4: Iteration:  600 / 2000 [ 30%]  (Warmup)
## Chain 4: Iteration:  800 / 2000 [ 40%]  (Warmup)
## Chain 4: Iteration: 1000 / 2000 [ 50%]  (Warmup)
## Chain 4: Iteration: 1001 / 2000 [ 50%]  (Sampling)
## Chain 4: Iteration: 1200 / 2000 [ 60%]  (Sampling)
## Chain 4: Iteration: 1400 / 2000 [ 70%]  (Sampling)
## Chain 4: Iteration: 1600 / 2000 [ 80%]  (Sampling)
## Chain 4: Iteration: 1800 / 2000 [ 90%]  (Sampling)
## Chain 4: Iteration: 2000 / 2000 [100%]  (Sampling)
## Chain 4: 
## Chain 4:  Elapsed Time: 0.06 seconds (Warm-up)
## Chain 4:                0.065 seconds (Sampling)
## Chain 4:                0.125 seconds (Total)
## Chain 4:
```

```
plot(fit_prot1_post, ask = FALSE)
```

```
summary(fit_prot1_post)
```

```
##  Family: gaussian 
##   Links: mu = identity; sigma = identity 
## Formula: abundance ~ 0 + type + time + (type:time) 
##    Data: data (Number of observations: 20) 
## Samples: 4 chains, each with iter = 2000; warmup = 1000; thin = 1;
##          total post-warmup samples = 4000
## 
## Population-Level Effects: 
##                  Estimate Est.Error l-95% CI u-95% CI Rhat Bulk_ESS Tail_ESS
## typeoops             0.08      0.06    -0.03     0.20 1.00     1645     1909
## typetotal            0.30      0.06     0.19     0.42 1.00     2009     1756
## time23              -0.28      0.08    -0.44    -0.12 1.00     1672     1775
## time6                0.07      0.07    -0.07     0.23 1.00     1650     2076
## typetotal:time23     0.13      0.11    -0.09     0.36 1.00     1625     2041
## typetotal:time6     -0.24      0.11    -0.45    -0.03 1.00     1524     1880
## 
## Family Specific Parameters: 
##       Estimate Est.Error l-95% CI u-95% CI Rhat Bulk_ESS Tail_ESS
## sigma     0.10      0.02     0.07     0.14 1.00     1881     2420
## 
## Samples were drawn using sampling(NUTS). For each parameter, Bulk_ESS
## and Tail_ESS are effective sample size measures, and Rhat is the potential
## scale reduction factor on split chains (at convergence, Rhat = 1).
```

Before, we dive into what the model is telling us we check that our Bayesian
computations have been faithful. Here `Rhat` is about \(1\) and we can assume
that it is unlikely there are any computational obstructions.

The above demonstrates the posterior distributions have clearly learnt something
from the data. Let us have a look at one of the distributions for the parameters.
The prior in orange is clearly spread out compared to the posterior distribution
in green. It looks like the data was very informative of this parameter.

```
hist(posterior_samples(fit_prot1)[,1],
     main = "Posterior Distribution type=oops",
     breaks = 20, col = brewer.pal(n = 3, name = "Set2")[2], xlab = "estimate")
hist(posterior_samples(fit_prot1_post)[,1],
     main = "Posterior Distribution type=oops",
     breaks = 30, col = brewer.pal(n = 3, name = "Set2")[1], xlab = "estimate", add = TRUE)
```

We can compute the posterior contraction to how much concentration there is of
the posterior. The value is close to 1 suggesting the parameters is well-informed
by the data.

```
contraction <- 1 - (sd(posterior_samples(fit_prot1_post)[,1])/sd(posterior_samples(fit_prot1)[,1]))
contraction
```

```
## [1] 0.9412167
```

Now let us have a look at the posterior predictive checks. From each of the plots
below we can see that the posterior predictive distribution matches the summaries
of the data extremely well. This suggests that data generated by using the
posterior estimates of the parameters look like the data we observed.

```
# compute kernel density estimate of data and simulated data and plot
pp_check(object = fit_prot1_post, "dens_overlay")
```

```
## Using 10 posterior samples for ppc type 'dens_overlay' by default.
```

```
# plot data in a histogram to see distributions
pp_check(object = fit_prot1_post, "hist")
```

```
## Using 10 posterior samples for ppc type 'hist' by default.
```

```
## `stat_bin()` using `bins = 30`. Pick better value with `binwidth`.
```

```
# plot data in a boxplot to see distribtuions
pp_check(object = fit_prot1_post, "boxplot")
```

```
## Using 10 posterior samples for ppc type 'boxplot' by default.
```

```
## notch went outside hinges. Try setting notch=FALSE.
## notch went outside hinges. Try setting notch=FALSE.
## notch went outside hinges. Try setting notch=FALSE.
```

```
# produce summary statistics
pp_check(object = fit_prot1_post, "stat_2d")
```

```
## Using all posterior samples for ppc type 'stat_2d' by default.
```

```
# produce intervals
pp_check(object = fit_prot1_post, "intervals")
```

```
## Using all posterior samples for ppc type 'intervals' by default.
```

```
# predctive errors
pp_check(object = fit_prot1_post, "error_hist")
```

```
## Using 10 posterior samples for ppc type 'error_hist' by default.
```

```
## `stat_bin()` using `bins = 30`. Pick better value with `binwidth`.
```

We notice that there is a small bump at the lower end of the first density plot.
We can generate more samples to see if this bump is something we can generate
from the data. From the plot below it shows we can generate similar to that
observed. However, there is a concern that simulated data is perhaps producing
data more extreme that that observed, as well as occasionally some unrealistic
looking data.

```
pp_check(object = fit_prot1_post, "dens_overlay", nsamples = 50)
```

# 6 Using uncertainty

Before we investigate more complex models, we examine what the
uncertainty can do for us to understand our model, inferences and problem.
Recall, we are particularly interested in the direction between type and time.
We first look at the interaction at 6 hours with type. As we can see from
the plot the estimate for the interaction is mostly negative. This suggests
that *relative* to the total sample the oops sample increased. This may
lead us to believe that the proprotion of the protein that is RNA-bound is
greater.

```
hist(posterior_samples(fit_prot1_post)[,6], 
     breaks = 30, col = brewer.pal(n = 3, name = "Set2")[1],
     xlab = "estimate", 
     main = "Posterior estimates for interaction between total and 6 h")
```

Since we are working with probability distributions, we can simply calculate
the probability that this estimate is less than 0. We see this is \(0.98525\) and
we can also compute other probabilities as well. For example, we may only
be interested in meaningful effect sizes.

```
# Probability less than 0
sum(posterior_samples(fit_prot1_post)[,6] < 0)/length(posterior_samples(fit_prot1_post)[,6])
```

```
## [1] 0.98575
```

```
# Probability less than -0.1
sum(posterior_samples(fit_prot1_post)[,6] < -0.1)/length(posterior_samples(fit_prot1_post)[,6])
```

```
## [1] 0.90475
```

We can also examine the interaction effect at 23h, where we see the effect is
in the opposite direction.

```
hist(posterior_samples(fit_prot1_post)[,5], 
     breaks = 30, col = brewer.pal(n = 3, name = "Set2")[2],
     xlab = "estimate", 
     main = "Posterior estimates for interaction between total and 23 h")
```

We can calculate probabilistic quantities from this distribution and we can
see the effect is much weaker in this case. However, there is still some evidence
for an decrease in the proportion of this protein binding RNA relative to total
protein at 23h post noczodole arrest.

```
# Probability greater than 0
sum(posterior_samples(fit_prot1_post)[,5] > 0)/length(posterior_samples(fit_prot1_post)[,5])
```

```
## [1] 0.889
```

```
# Probability greater than -0.1
sum(posterior_samples(fit_prot1_post)[,5] > 0.1)/length(posterior_samples(fit_prot1_post)[,5])
```

```
## [1] 0.61875
```

We can also ask more elaborate probabilistic questions of these data. We can
ask the probability that the changes for the interaction terms
were in the same direction at 6 hours and 23 hours. We see that the probability
that the effect is in same direction is quite small and more likely the effects
are in different directions.

```
probBothPositive <- sum(posterior_samples(fit_prot1_post)[,5] > 0 & 
                            posterior_samples(fit_prot1_post)[,6] > 0)/4000
probBothNegative <- sum(posterior_samples(fit_prot1_post)[,5] < 0 & 
                            posterior_samples(fit_prot1_post)[,6] < 0)/4000
probBothNegative + probBothPositive
```

```
## [1] 0.12525
```

We can also compute the probability that the effects are greater than \(0.1\) at
both times in any direction. We can see we are quite uncertain as to whether
the effects are both simultaneously greater than \(0.1\). Of course we can ask
more complex probability questions in the same manner.

```
sum(abs(posterior_samples(fit_prot1_post)[,5]) > 0.1 & 
        abs(posterior_samples(fit_prot1_post)[,6]) > 0.1)/4000
```

```
## [1] 0.555
```

Credible interval can be obtained from the summary.

```
summary(fit_prot1_post)
```

```
##  Family: gaussian 
##   Links: mu = identity; sigma = identity 
## Formula: abundance ~ 0 + type + time + (type:time) 
##    Data: data (Number of observations: 20) 
## Samples: 4 chains, each with iter = 2000; warmup = 1000; thin = 1;
##          total post-warmup samples = 4000
## 
## Population-Level Effects: 
##                  Estimate Est.Error l-95% CI u-95% CI Rhat Bulk_ESS Tail_ESS
## typeoops             0.08      0.06    -0.03     0.20 1.00     1645     1909
## typetotal            0.30      0.06     0.19     0.42 1.00     2009     1756
## time23              -0.28      0.08    -0.44    -0.12 1.00     1672     1775
## time6                0.07      0.07    -0.07     0.23 1.00     1650     2076
## typetotal:time23     0.13      0.11    -0.09     0.36 1.00     1625     2041
## typetotal:time6     -0.24      0.11    -0.45    -0.03 1.00     1524     1880
## 
## Family Specific Parameters: 
##       Estimate Est.Error l-95% CI u-95% CI Rhat Bulk_ESS Tail_ESS
## sigma     0.10      0.02     0.07     0.14 1.00     1881     2420
## 
## Samples were drawn using sampling(NUTS). For each parameter, Bulk_ESS
## and Tail_ESS are effective sample size measures, and Rhat is the potential
## scale reduction factor on split chains (at convergence, Rhat = 1).
```

This provides the basics of the workflow. We have a probabilistic model
of which we can ask questions and quantify our uncertainties.
