# Supplementary material for: Challenges and Opportunities for Bayesian Statistics in Proteomics
Source: J Proteome Res. 2022 Mar 8;21(4):849–64. doi: 10.1021/acs.jproteome.1c00859 (PMC8982455; doi:10.1021/acs.jproteome.1c00859)
Supplement: Supplementary file 2 — pr1c00859_si_002.zip [file pr1c00859_si_002.zip › Supporting information for publication/oops_modelling_part2.html]

Bayesian analysis of Organic Orthogonal Phase Seperation Data Part 2


# Bayesian analysis of Organic Orthogonal Phase Seperation Data Part 2

Oliver M. Crook

#### 22/09/2021

# 1 Introduction

Here, we attempt to build a more advanced principled Bayesian model
for some OOPS data. We illustrate the main ideas of a Bayesian workflow and refer to the
accompanying manuscript for relevant details. The first code chunk loads the
packages we are interested in.

```
library(brms)
```

```
## Loading required package: Rcpp
```

```
## Loading 'brms' package (version 2.16.1). Useful instructions
## can be found by typing help('brms'). A more detailed introduction
## to the package is available through vignette('brms_overview').
```

```
## 
## Attaching package: 'brms'
```

```
## The following object is masked from 'package:stats':
## 
##     ar
```

```
library(pheatmap)
library(RColorBrewer)
library(MSnbase)
```

```
## Loading required package: BiocGenerics
```

```
## Loading required package: parallel
```

```
## 
## Attaching package: 'BiocGenerics'
```

```
## The following objects are masked from 'package:parallel':
## 
##     clusterApply, clusterApplyLB, clusterCall, clusterEvalQ,
##     clusterExport, clusterMap, parApply, parCapply, parLapply,
##     parLapplyLB, parRapply, parSapply, parSapplyLB
```

```
## The following objects are masked from 'package:stats':
## 
##     IQR, mad, sd, var, xtabs
```

```
## The following objects are masked from 'package:base':
## 
##     anyDuplicated, append, as.data.frame, basename, cbind, colnames,
##     dirname, do.call, duplicated, eval, evalq, Filter, Find, get, grep,
##     grepl, intersect, is.unsorted, lapply, Map, mapply, match, mget,
##     order, paste, pmax, pmax.int, pmin, pmin.int, Position, rank,
##     rbind, Reduce, rownames, sapply, setdiff, sort, table, tapply,
##     union, unique, unsplit, which.max, which.min
```

```
## Loading required package: Biobase
```

```
## Welcome to Bioconductor
## 
##     Vignettes contain introductory material; view with
##     'browseVignettes()'. To cite Bioconductor, see
##     'citation("Biobase")', and for packages 'citation("pkgname")'.
```

```
## Loading required package: mzR
```

```
## Warning in fun(libname, pkgname): mzR has been built against a different Rcpp version (1.0.6)
## than is installed on your system (1.0.7). This might lead to errors
## when loading mzR. If you encounter such issues, please send a report,
## including the output of sessionInfo() to the Bioc support forum at 
## https://support.bioconductor.org/. For details see also
## https://github.com/sneumann/mzR/wiki/mzR-Rcpp-compiler-linker-issue.
```

```
## Loading required package: S4Vectors
```

```
## Loading required package: stats4
```

```
## 
## Attaching package: 'S4Vectors'
```

```
## The following objects are masked from 'package:base':
## 
##     expand.grid, I, unname
```

```
## Loading required package: ProtGenerics
```

```
## 
## Attaching package: 'ProtGenerics'
```

```
## The following object is masked from 'package:stats':
## 
##     smooth
```

```
## Warning: multiple methods tables found for 'bin'
```

```
## Warning: multiple methods tables found for 'compareSpectra'
```

```
## 
## This is MSnbase version 2.18.0 
##   Visit https://lgatto.github.io/MSnbase/ to get started.
```

```
## 
## Attaching package: 'MSnbase'
```

```
## The following objects are masked from 'package:ProtGenerics':
## 
##     bin, compareSpectra
```

```
## The following object is masked from 'package:base':
## 
##     trimws
```

```
library(ggplot2)
library(ggfortify)
bayesplot::color_scheme_set("teal")
```

Now, we load the data we are interested, which is from Queiroz et al. 2019.
The data have already been summarised to protein level and column median normalised.
The design used 2 seperate TMT 10-plexes to measure protein abundance and oops sample. This
allows us to model changes in RNA binding that are indepedent of concurrent changes
in total protien abundance. Changes in oops-enriched protein abundance samples
that do not correlated with total protein abundance chances are indicative
of specific RBPs binding RNA differentially in those conditions. The experimental
design uses thymidine-nocodazole arrest and then measures protein abundance,
as well as oops enriched protein abundance at \(0,6\) and \(23\) hours. Triplicate
measurements are made, except for at 6 hours where 4 replicates where taken.
For further details see Queiroz et al. 2019

```
# We assume the data is in the working directory
oopsdata <- readMSnSet2(file = "oopsdata.csv", ecol = 2:21, fnames = 1)
```

We test using a single protein for simplicity

```
set.seed(1)
data <- data.frame(exprs(oopsdata)[sample.int(nrow(oopsdata), size = 1),])
colnames(data) <- "abundance"
data$type <- as.factor(rep(c("total", "oops"), each = 10))
data$time <- as.factor(rep(rep(c("0", "6", "23"), times = c(3,4,3)), 2))
data$replicate <- as.factor(sapply(strsplit(rownames(data), "_"), function(x)x[2]))
data$tag <- as.factor(rep(TMT10@reporterNames, times = 2))
```

# 2 More complex modelling, allowing the noise to vary between samples

We previously left aside some of the modelling aspects to explore the full
workflow. Let us explore building and evaluating a more bespoke model, that
captures more of the variations in the data. The first point we raised was
that the variance was different for the oops samples, total samples and at
different times. In the following we allow \(\sigma\) to vary with sample type
and time. Note we are now on the log scale and so move to a normal prior so that
\[
\log \sigma = \beta\_{type} + \beta\_{time}\\
\beta \sim \mathcal{N}(0, 3).
\]

```
pr <- c(prior(normal(0,1), class = "b"),
  prior(normal(0, 3), dpar = "sigma"))


fit_prot1_post2 <- brm(bf(abundance ~ 0 + type + time + (type:time),
                     sigma ~ 0 + type + time),
                 data = data,
                 family = gaussian(link = "identity"),
                 sample_prior = "no",
                 prior = pr)
```

```
## Compiling Stan program...
```

```
## Start sampling
```

```
## 
## SAMPLING FOR MODEL '05945ccf0907ef96ed16640d6a083913' NOW (CHAIN 1).
## Chain 1: 
## Chain 1: Gradient evaluation took 0 seconds
## Chain 1: 1000 transitions using 10 leapfrog steps per transition would take 0 seconds.
## Chain 1: Adjust your expectations accordingly!
## Chain 1: 
## Chain 1: 
## Chain 1: Iteration:    1 / 2000 [  0%]  (Warmup)
## Chain 1: Iteration:  200 / 2000 [ 10%]  (Warmup)
## Chain 1: Iteration:  400 / 2000 [ 20%]  (Warmup)
## Chain 1: Iteration:  600 / 2000 [ 30%]  (Warmup)
## Chain 1: Iteration:  800 / 2000 [ 40%]  (Warmup)
## Chain 1: Iteration: 1000 / 2000 [ 50%]  (Warmup)
## Chain 1: Iteration: 1001 / 2000 [ 50%]  (Sampling)
## Chain 1: Iteration: 1200 / 2000 [ 60%]  (Sampling)
## Chain 1: Iteration: 1400 / 2000 [ 70%]  (Sampling)
## Chain 1: Iteration: 1600 / 2000 [ 80%]  (Sampling)
## Chain 1: Iteration: 1800 / 2000 [ 90%]  (Sampling)
## Chain 1: Iteration: 2000 / 2000 [100%]  (Sampling)
## Chain 1: 
## Chain 1:  Elapsed Time: 0.267 seconds (Warm-up)
## Chain 1:                0.245 seconds (Sampling)
## Chain 1:                0.512 seconds (Total)
## Chain 1: 
## 
## SAMPLING FOR MODEL '05945ccf0907ef96ed16640d6a083913' NOW (CHAIN 2).
## Chain 2: 
## Chain 2: Gradient evaluation took 0 seconds
## Chain 2: 1000 transitions using 10 leapfrog steps per transition would take 0 seconds.
## Chain 2: Adjust your expectations accordingly!
## Chain 2: 
## Chain 2: 
## Chain 2: Iteration:    1 / 2000 [  0%]  (Warmup)
## Chain 2: Iteration:  200 / 2000 [ 10%]  (Warmup)
## Chain 2: Iteration:  400 / 2000 [ 20%]  (Warmup)
## Chain 2: Iteration:  600 / 2000 [ 30%]  (Warmup)
## Chain 2: Iteration:  800 / 2000 [ 40%]  (Warmup)
## Chain 2: Iteration: 1000 / 2000 [ 50%]  (Warmup)
## Chain 2: Iteration: 1001 / 2000 [ 50%]  (Sampling)
## Chain 2: Iteration: 1200 / 2000 [ 60%]  (Sampling)
## Chain 2: Iteration: 1400 / 2000 [ 70%]  (Sampling)
## Chain 2: Iteration: 1600 / 2000 [ 80%]  (Sampling)
## Chain 2: Iteration: 1800 / 2000 [ 90%]  (Sampling)
## Chain 2: Iteration: 2000 / 2000 [100%]  (Sampling)
## Chain 2: 
## Chain 2:  Elapsed Time: 0.296 seconds (Warm-up)
## Chain 2:                0.245 seconds (Sampling)
## Chain 2:                0.541 seconds (Total)
## Chain 2: 
## 
## SAMPLING FOR MODEL '05945ccf0907ef96ed16640d6a083913' NOW (CHAIN 3).
## Chain 3: 
## Chain 3: Gradient evaluation took 0 seconds
## Chain 3: 1000 transitions using 10 leapfrog steps per transition would take 0 seconds.
## Chain 3: Adjust your expectations accordingly!
## Chain 3: 
## Chain 3: 
## Chain 3: Iteration:    1 / 2000 [  0%]  (Warmup)
## Chain 3: Iteration:  200 / 2000 [ 10%]  (Warmup)
## Chain 3: Iteration:  400 / 2000 [ 20%]  (Warmup)
## Chain 3: Iteration:  600 / 2000 [ 30%]  (Warmup)
## Chain 3: Iteration:  800 / 2000 [ 40%]  (Warmup)
## Chain 3: Iteration: 1000 / 2000 [ 50%]  (Warmup)
## Chain 3: Iteration: 1001 / 2000 [ 50%]  (Sampling)
## Chain 3: Iteration: 1200 / 2000 [ 60%]  (Sampling)
## Chain 3: Iteration: 1400 / 2000 [ 70%]  (Sampling)
## Chain 3: Iteration: 1600 / 2000 [ 80%]  (Sampling)
## Chain 3: Iteration: 1800 / 2000 [ 90%]  (Sampling)
## Chain 3: Iteration: 2000 / 2000 [100%]  (Sampling)
## Chain 3: 
## Chain 3:  Elapsed Time: 0.275 seconds (Warm-up)
## Chain 3:                0.197 seconds (Sampling)
## Chain 3:                0.472 seconds (Total)
## Chain 3: 
## 
## SAMPLING FOR MODEL '05945ccf0907ef96ed16640d6a083913' NOW (CHAIN 4).
## Chain 4: 
## Chain 4: Gradient evaluation took 0 seconds
## Chain 4: 1000 transitions using 10 leapfrog steps per transition would take 0 seconds.
## Chain 4: Adjust your expectations accordingly!
## Chain 4: 
## Chain 4: 
## Chain 4: Iteration:    1 / 2000 [  0%]  (Warmup)
## Chain 4: Iteration:  200 / 2000 [ 10%]  (Warmup)
## Chain 4: Iteration:  400 / 2000 [ 20%]  (Warmup)
## Chain 4: Iteration:  600 / 2000 [ 30%]  (Warmup)
## Chain 4: Iteration:  800 / 2000 [ 40%]  (Warmup)
## Chain 4: Iteration: 1000 / 2000 [ 50%]  (Warmup)
## Chain 4: Iteration: 1001 / 2000 [ 50%]  (Sampling)
## Chain 4: Iteration: 1200 / 2000 [ 60%]  (Sampling)
## Chain 4: Iteration: 1400 / 2000 [ 70%]  (Sampling)
## Chain 4: Iteration: 1600 / 2000 [ 80%]  (Sampling)
## Chain 4: Iteration: 1800 / 2000 [ 90%]  (Sampling)
## Chain 4: Iteration: 2000 / 2000 [100%]  (Sampling)
## Chain 4: 
## Chain 4:  Elapsed Time: 0.245 seconds (Warm-up)
## Chain 4:                0.183 seconds (Sampling)
## Chain 4:                0.428 seconds (Total)
## Chain 4:
```

```
plot(fit_prot1_post2, ask = FALSE)
```

```
summary(fit_prot1_post2)
```

```
##  Family: gaussian 
##   Links: mu = identity; sigma = log 
## Formula: abundance ~ 0 + type + time + (type:time) 
##          sigma ~ 0 + type + time
##    Data: data (Number of observations: 20) 
##   Draws: 4 chains, each with iter = 2000; warmup = 1000; thin = 1;
##          total post-warmup draws = 4000
## 
## Population-Level Effects: 
##                  Estimate Est.Error l-95% CI u-95% CI Rhat Bulk_ESS Tail_ESS
## typeoops             0.08      0.05    -0.02     0.18 1.00     1465     1332
## typetotal            0.30      0.04     0.21     0.38 1.00     1951     1227
## time23              -0.28      0.13    -0.56    -0.02 1.01     1468     1476
## time6                0.07      0.06    -0.04     0.20 1.00     1422     1415
## typetotal:time23     0.13      0.17    -0.21     0.51 1.00     1449     1235
## typetotal:time6     -0.24      0.08    -0.41    -0.07 1.00     1384     1514
## sigma_typeoops      -2.69      0.52    -3.55    -1.51 1.00      977     1107
## sigma_typetotal     -2.84      0.46    -3.56    -1.74 1.00      912     1003
## sigma_time23         0.97      0.58    -0.22     2.06 1.00     1117     1241
## sigma_time6          0.04      0.55    -1.13     1.05 1.00     1026      966
## 
## Draws were sampled using sampling(NUTS). For each parameter, Bulk_ESS
## and Tail_ESS are effective sample size measures, and Rhat is the potential
## scale reduction factor on split chains (at convergence, Rhat = 1).
```

We notice that the estimated errors for some of the effects have become larger
in this more complex example. The more complex model has become harder
to estimate and therefore our inferences are likely to be unreliable. The first
remedy is to provide stronger prior information. We already saw in previous
modelling that our priors we quite diffuse. Here, we place even stronger
prior information. We also proceed to perform posterior predictive checks as
before.

```
pr <- c(prior(normal(0,1), class = "b"),
  prior(normal(0,1), dpar = "sigma"))


fit_prot1_post2a <- brm(bf(abundance ~ 0 + type + time + (type:time),
                     sigma ~ 0 + type + time),
                 data = data,
                 family = gaussian(link = "identity"),
                 sample_prior = "no",
                 prior = pr,
                 control = list(adapt_delta = 0.99))
```

```
## Compiling Stan program...
```

```
## Start sampling
```

```
## 
## SAMPLING FOR MODEL 'de099b553a70635d6a8819683968b4e6' NOW (CHAIN 1).
## Chain 1: 
## Chain 1: Gradient evaluation took 0 seconds
## Chain 1: 1000 transitions using 10 leapfrog steps per transition would take 0 seconds.
## Chain 1: Adjust your expectations accordingly!
## Chain 1: 
## Chain 1: 
## Chain 1: Iteration:    1 / 2000 [  0%]  (Warmup)
## Chain 1: Iteration:  200 / 2000 [ 10%]  (Warmup)
## Chain 1: Iteration:  400 / 2000 [ 20%]  (Warmup)
## Chain 1: Iteration:  600 / 2000 [ 30%]  (Warmup)
## Chain 1: Iteration:  800 / 2000 [ 40%]  (Warmup)
## Chain 1: Iteration: 1000 / 2000 [ 50%]  (Warmup)
## Chain 1: Iteration: 1001 / 2000 [ 50%]  (Sampling)
## Chain 1: Iteration: 1200 / 2000 [ 60%]  (Sampling)
## Chain 1: Iteration: 1400 / 2000 [ 70%]  (Sampling)
## Chain 1: Iteration: 1600 / 2000 [ 80%]  (Sampling)
## Chain 1: Iteration: 1800 / 2000 [ 90%]  (Sampling)
## Chain 1: Iteration: 2000 / 2000 [100%]  (Sampling)
## Chain 1: 
## Chain 1:  Elapsed Time: 0.897 seconds (Warm-up)
## Chain 1:                0.831 seconds (Sampling)
## Chain 1:                1.728 seconds (Total)
## Chain 1: 
## 
## SAMPLING FOR MODEL 'de099b553a70635d6a8819683968b4e6' NOW (CHAIN 2).
## Chain 2: 
## Chain 2: Gradient evaluation took 0 seconds
## Chain 2: 1000 transitions using 10 leapfrog steps per transition would take 0 seconds.
## Chain 2: Adjust your expectations accordingly!
## Chain 2: 
## Chain 2: 
## Chain 2: Iteration:    1 / 2000 [  0%]  (Warmup)
## Chain 2: Iteration:  200 / 2000 [ 10%]  (Warmup)
## Chain 2: Iteration:  400 / 2000 [ 20%]  (Warmup)
## Chain 2: Iteration:  600 / 2000 [ 30%]  (Warmup)
## Chain 2: Iteration:  800 / 2000 [ 40%]  (Warmup)
## Chain 2: Iteration: 1000 / 2000 [ 50%]  (Warmup)
## Chain 2: Iteration: 1001 / 2000 [ 50%]  (Sampling)
## Chain 2: Iteration: 1200 / 2000 [ 60%]  (Sampling)
## Chain 2: Iteration: 1400 / 2000 [ 70%]  (Sampling)
## Chain 2: Iteration: 1600 / 2000 [ 80%]  (Sampling)
## Chain 2: Iteration: 1800 / 2000 [ 90%]  (Sampling)
## Chain 2: Iteration: 2000 / 2000 [100%]  (Sampling)
## Chain 2: 
## Chain 2:  Elapsed Time: 0.866 seconds (Warm-up)
## Chain 2:                0.742 seconds (Sampling)
## Chain 2:                1.608 seconds (Total)
## Chain 2: 
## 
## SAMPLING FOR MODEL 'de099b553a70635d6a8819683968b4e6' NOW (CHAIN 3).
## Chain 3: 
## Chain 3: Gradient evaluation took 0 seconds
## Chain 3: 1000 transitions using 10 leapfrog steps per transition would take 0 seconds.
## Chain 3: Adjust your expectations accordingly!
## Chain 3: 
## Chain 3: 
## Chain 3: Iteration:    1 / 2000 [  0%]  (Warmup)
## Chain 3: Iteration:  200 / 2000 [ 10%]  (Warmup)
## Chain 3: Iteration:  400 / 2000 [ 20%]  (Warmup)
## Chain 3: Iteration:  600 / 2000 [ 30%]  (Warmup)
## Chain 3: Iteration:  800 / 2000 [ 40%]  (Warmup)
## Chain 3: Iteration: 1000 / 2000 [ 50%]  (Warmup)
## Chain 3: Iteration: 1001 / 2000 [ 50%]  (Sampling)
## Chain 3: Iteration: 1200 / 2000 [ 60%]  (Sampling)
## Chain 3: Iteration: 1400 / 2000 [ 70%]  (Sampling)
## Chain 3: Iteration: 1600 / 2000 [ 80%]  (Sampling)
## Chain 3: Iteration: 1800 / 2000 [ 90%]  (Sampling)
## Chain 3: Iteration: 2000 / 2000 [100%]  (Sampling)
## Chain 3: 
## Chain 3:  Elapsed Time: 0.778 seconds (Warm-up)
## Chain 3:                0.586 seconds (Sampling)
## Chain 3:                1.364 seconds (Total)
## Chain 3: 
## 
## SAMPLING FOR MODEL 'de099b553a70635d6a8819683968b4e6' NOW (CHAIN 4).
## Chain 4: 
## Chain 4: Gradient evaluation took 0 seconds
## Chain 4: 1000 transitions using 10 leapfrog steps per transition would take 0 seconds.
## Chain 4: Adjust your expectations accordingly!
## Chain 4: 
## Chain 4: 
## Chain 4: Iteration:    1 / 2000 [  0%]  (Warmup)
## Chain 4: Iteration:  200 / 2000 [ 10%]  (Warmup)
## Chain 4: Iteration:  400 / 2000 [ 20%]  (Warmup)
## Chain 4: Iteration:  600 / 2000 [ 30%]  (Warmup)
## Chain 4: Iteration:  800 / 2000 [ 40%]  (Warmup)
## Chain 4: Iteration: 1000 / 2000 [ 50%]  (Warmup)
## Chain 4: Iteration: 1001 / 2000 [ 50%]  (Sampling)
## Chain 4: Iteration: 1200 / 2000 [ 60%]  (Sampling)
## Chain 4: Iteration: 1400 / 2000 [ 70%]  (Sampling)
## Chain 4: Iteration: 1600 / 2000 [ 80%]  (Sampling)
## Chain 4: Iteration: 1800 / 2000 [ 90%]  (Sampling)
## Chain 4: Iteration: 2000 / 2000 [100%]  (Sampling)
## Chain 4: 
## Chain 4:  Elapsed Time: 0.861 seconds (Warm-up)
## Chain 4:                1.192 seconds (Sampling)
## Chain 4:                2.053 seconds (Total)
## Chain 4:
```

```
plot(fit_prot1_post2a, ask = FALSE)
```

```
summary(fit_prot1_post2a)
```

```
##  Family: gaussian 
##   Links: mu = identity; sigma = log 
## Formula: abundance ~ 0 + type + time + (type:time) 
##          sigma ~ 0 + type + time
##    Data: data (Number of observations: 20) 
##   Draws: 4 chains, each with iter = 2000; warmup = 1000; thin = 1;
##          total post-warmup draws = 4000
## 
## Population-Level Effects: 
##                  Estimate Est.Error l-95% CI u-95% CI Rhat Bulk_ESS Tail_ESS
## typeoops             0.08      0.11    -0.17     0.31 1.01      964     1004
## typetotal            0.31      0.10     0.11     0.52 1.00     1362     1056
## time23              -0.27      0.16    -0.59     0.04 1.00     1157     1198
## time6                0.08      0.12    -0.17     0.33 1.00      946      970
## typetotal:time23     0.12      0.21    -0.30     0.54 1.00     1096     1305
## typetotal:time6     -0.25      0.16    -0.57     0.06 1.00      868      932
## sigma_typeoops      -1.83      0.51    -2.78    -0.80 1.00      888     1880
## sigma_typetotal     -2.03      0.48    -2.92    -1.02 1.00      843     1513
## sigma_time23         0.11      0.52    -0.90     1.12 1.00     1059     1808
## sigma_time6         -0.73      0.50    -1.71     0.29 1.00     1007     2061
## 
## Draws were sampled using sampling(NUTS). For each parameter, Bulk_ESS
## and Tail_ESS are effective sample size measures, and Rhat is the potential
## scale reduction factor on split chains (at convergence, Rhat = 1).
```

```
# compute kernel density estimate of data and simulated data and plot
pp_check(object = fit_prot1_post2a, "dens_overlay")
```

```
## Using 10 posterior draws for ppc type 'dens_overlay' by default.
```

```
# plot data in a histogram to see distributions
pp_check(object = fit_prot1_post2a, "hist")
```

```
## Using 10 posterior draws for ppc type 'hist' by default.
```

```
## `stat_bin()` using `bins = 30`. Pick better value with `binwidth`.
```

```
# plot data in a boxplot to see distribtuions
pp_check(object = fit_prot1_post2a, "boxplot")
```

```
## Using 10 posterior draws for ppc type 'boxplot' by default.
```

```
## notch went outside hinges. Try setting notch=FALSE.
## notch went outside hinges. Try setting notch=FALSE.
## notch went outside hinges. Try setting notch=FALSE.
```

```
# produce summary statistics
pp_check(object = fit_prot1_post2a, "stat_2d")
```

```
## Using all posterior draws for ppc type 'stat_2d' by default.
```

```
# produce intervals
pp_check(object = fit_prot1_post2a, "intervals")
```

```
## Using all posterior draws for ppc type 'intervals' by default.
```

```
# predctive errors
pp_check(object = fit_prot1_post2a, "error_hist")
```

```
## Using 10 posterior draws for ppc type 'error_hist' by default.
```

```
## `stat_bin()` using `bins = 30`. Pick better value with `binwidth`.
```

# 3 Using a heavy tailed prior

We still see that the estimated errors are quite large for the data with excessive
mass in the extremes of the distribution. We need to think
more carefully about the model we are trying to fit. The normal prior
does not allow much variation in the scale of \(\sigma\) and given that we are
now allowing the standard deviations to vary between samples our confidence
is the scale is more uncertain. Our current modelling approach has not captured
this and so we need to use a prior that reflects this uncertainty. We can do this
using a heavy tailed prior on \(\sigma\). We opt for a student-t prior as
below. The heavy tails also induce more shrinkage of the coeffcients towards \(0\).

```
pr <- c(prior(normal(0, 1), class = "b"),
  prior(student_t(3, 0, 1), dpar = "sigma"))


fit_prot1_post2b <- brm(bf(abundance ~ 0 + type + time + (type:time),
                     sigma ~ 0 + type + time),
                 data = data,
                 family = gaussian(link = "identity"),
                 sample_prior = "no",
                 prior = pr,
                 control = list(adapt_delta = 0.99),
                 save_pars = save_pars(all = TRUE))
```

```
## Compiling Stan program...
```

```
## Start sampling
```

```
## 
## SAMPLING FOR MODEL '23ba2da0424b898192de662793768584' NOW (CHAIN 1).
## Chain 1: 
## Chain 1: Gradient evaluation took 0 seconds
## Chain 1: 1000 transitions using 10 leapfrog steps per transition would take 0 seconds.
## Chain 1: Adjust your expectations accordingly!
## Chain 1: 
## Chain 1: 
## Chain 1: Iteration:    1 / 2000 [  0%]  (Warmup)
## Chain 1: Iteration:  200 / 2000 [ 10%]  (Warmup)
## Chain 1: Iteration:  400 / 2000 [ 20%]  (Warmup)
## Chain 1: Iteration:  600 / 2000 [ 30%]  (Warmup)
## Chain 1: Iteration:  800 / 2000 [ 40%]  (Warmup)
## Chain 1: Iteration: 1000 / 2000 [ 50%]  (Warmup)
## Chain 1: Iteration: 1001 / 2000 [ 50%]  (Sampling)
## Chain 1: Iteration: 1200 / 2000 [ 60%]  (Sampling)
## Chain 1: Iteration: 1400 / 2000 [ 70%]  (Sampling)
## Chain 1: Iteration: 1600 / 2000 [ 80%]  (Sampling)
## Chain 1: Iteration: 1800 / 2000 [ 90%]  (Sampling)
## Chain 1: Iteration: 2000 / 2000 [100%]  (Sampling)
## Chain 1: 
## Chain 1:  Elapsed Time: 0.826 seconds (Warm-up)
## Chain 1:                0.523 seconds (Sampling)
## Chain 1:                1.349 seconds (Total)
## Chain 1: 
## 
## SAMPLING FOR MODEL '23ba2da0424b898192de662793768584' NOW (CHAIN 2).
## Chain 2: 
## Chain 2: Gradient evaluation took 0 seconds
## Chain 2: 1000 transitions using 10 leapfrog steps per transition would take 0 seconds.
## Chain 2: Adjust your expectations accordingly!
## Chain 2: 
## Chain 2: 
## Chain 2: Iteration:    1 / 2000 [  0%]  (Warmup)
## Chain 2: Iteration:  200 / 2000 [ 10%]  (Warmup)
## Chain 2: Iteration:  400 / 2000 [ 20%]  (Warmup)
## Chain 2: Iteration:  600 / 2000 [ 30%]  (Warmup)
## Chain 2: Iteration:  800 / 2000 [ 40%]  (Warmup)
## Chain 2: Iteration: 1000 / 2000 [ 50%]  (Warmup)
## Chain 2: Iteration: 1001 / 2000 [ 50%]  (Sampling)
## Chain 2: Iteration: 1200 / 2000 [ 60%]  (Sampling)
## Chain 2: Iteration: 1400 / 2000 [ 70%]  (Sampling)
## Chain 2: Iteration: 1600 / 2000 [ 80%]  (Sampling)
## Chain 2: Iteration: 1800 / 2000 [ 90%]  (Sampling)
## Chain 2: Iteration: 2000 / 2000 [100%]  (Sampling)
## Chain 2: 
## Chain 2:  Elapsed Time: 0.677 seconds (Warm-up)
## Chain 2:                0.859 seconds (Sampling)
## Chain 2:                1.536 seconds (Total)
## Chain 2: 
## 
## SAMPLING FOR MODEL '23ba2da0424b898192de662793768584' NOW (CHAIN 3).
## Chain 3: 
## Chain 3: Gradient evaluation took 0 seconds
## Chain 3: 1000 transitions using 10 leapfrog steps per transition would take 0 seconds.
## Chain 3: Adjust your expectations accordingly!
## Chain 3: 
## Chain 3: 
## Chain 3: Iteration:    1 / 2000 [  0%]  (Warmup)
## Chain 3: Iteration:  200 / 2000 [ 10%]  (Warmup)
## Chain 3: Iteration:  400 / 2000 [ 20%]  (Warmup)
## Chain 3: Iteration:  600 / 2000 [ 30%]  (Warmup)
## Chain 3: Iteration:  800 / 2000 [ 40%]  (Warmup)
## Chain 3: Iteration: 1000 / 2000 [ 50%]  (Warmup)
## Chain 3: Iteration: 1001 / 2000 [ 50%]  (Sampling)
## Chain 3: Iteration: 1200 / 2000 [ 60%]  (Sampling)
## Chain 3: Iteration: 1400 / 2000 [ 70%]  (Sampling)
## Chain 3: Iteration: 1600 / 2000 [ 80%]  (Sampling)
## Chain 3: Iteration: 1800 / 2000 [ 90%]  (Sampling)
## Chain 3: Iteration: 2000 / 2000 [100%]  (Sampling)
## Chain 3: 
## Chain 3:  Elapsed Time: 0.578 seconds (Warm-up)
## Chain 3:                0.591 seconds (Sampling)
## Chain 3:                1.169 seconds (Total)
## Chain 3: 
## 
## SAMPLING FOR MODEL '23ba2da0424b898192de662793768584' NOW (CHAIN 4).
## Chain 4: 
## Chain 4: Gradient evaluation took 0 seconds
## Chain 4: 1000 transitions using 10 leapfrog steps per transition would take 0 seconds.
## Chain 4: Adjust your expectations accordingly!
## Chain 4: 
## Chain 4: 
## Chain 4: Iteration:    1 / 2000 [  0%]  (Warmup)
## Chain 4: Iteration:  200 / 2000 [ 10%]  (Warmup)
## Chain 4: Iteration:  400 / 2000 [ 20%]  (Warmup)
## Chain 4: Iteration:  600 / 2000 [ 30%]  (Warmup)
## Chain 4: Iteration:  800 / 2000 [ 40%]  (Warmup)
## Chain 4: Iteration: 1000 / 2000 [ 50%]  (Warmup)
## Chain 4: Iteration: 1001 / 2000 [ 50%]  (Sampling)
## Chain 4: Iteration: 1200 / 2000 [ 60%]  (Sampling)
## Chain 4: Iteration: 1400 / 2000 [ 70%]  (Sampling)
## Chain 4: Iteration: 1600 / 2000 [ 80%]  (Sampling)
## Chain 4: Iteration: 1800 / 2000 [ 90%]  (Sampling)
## Chain 4: Iteration: 2000 / 2000 [100%]  (Sampling)
## Chain 4: 
## Chain 4:  Elapsed Time: 0.61 seconds (Warm-up)
## Chain 4:                0.722 seconds (Sampling)
## Chain 4:                1.332 seconds (Total)
## Chain 4:
```

```
plot(fit_prot1_post2b, ask = FALSE)
```

```
summary(fit_prot1_post2b)
```

```
##  Family: gaussian 
##   Links: mu = identity; sigma = log 
## Formula: abundance ~ 0 + type + time + (type:time) 
##          sigma ~ 0 + type + time
##    Data: data (Number of observations: 20) 
##   Draws: 4 chains, each with iter = 2000; warmup = 1000; thin = 1;
##          total post-warmup draws = 4000
## 
## Population-Level Effects: 
##                  Estimate Est.Error l-95% CI u-95% CI Rhat Bulk_ESS Tail_ESS
## typeoops             0.08      0.09    -0.10     0.26 1.01     1027      963
## typetotal            0.30      0.07     0.17     0.44 1.00     1521     1261
## time23              -0.27      0.15    -0.56     0.04 1.00     1356     1396
## time6                0.08      0.10    -0.11     0.27 1.01     1066      980
## typetotal:time23     0.11      0.19    -0.27     0.47 1.00     1376     1308
## typetotal:time6     -0.25      0.12    -0.50    -0.00 1.01     1036     1010
## sigma_typeoops      -2.24      0.56    -3.19    -1.04 1.01      703     1036
## sigma_typetotal     -2.44      0.54    -3.31    -1.27 1.01      708     1023
## sigma_time23         0.48      0.56    -0.65     1.52 1.01      934     1209
## sigma_time6         -0.36      0.54    -1.52     0.63 1.01      821     1491
## 
## Draws were sampled using sampling(NUTS). For each parameter, Bulk_ESS
## and Tail_ESS are effective sample size measures, and Rhat is the potential
## scale reduction factor on split chains (at convergence, Rhat = 1).
```

We can again perform posterior predictive checks to see if our model has
good predictive qualities. We can visually see that the model is capturing
the behaviour of the data extremely well. Though it probably be reasonable
to provide even stronger prior information.

```
# compute kernel density estimate of data and simulated data and plot
pp_check(object = fit_prot1_post2b, "dens_overlay")
```

```
## Using 10 posterior draws for ppc type 'dens_overlay' by default.
```

```
# plot data in a histogram to see distributions
pp_check(object = fit_prot1_post2b, "hist")
```

```
## Using 10 posterior draws for ppc type 'hist' by default.
```

```
## `stat_bin()` using `bins = 30`. Pick better value with `binwidth`.
```

```
# plot data in a boxplot to see distribtuions
pp_check(object = fit_prot1_post2b, "boxplot")
```

```
## Using 10 posterior draws for ppc type 'boxplot' by default.
```

```
## notch went outside hinges. Try setting notch=FALSE.
## notch went outside hinges. Try setting notch=FALSE.
## notch went outside hinges. Try setting notch=FALSE.
## notch went outside hinges. Try setting notch=FALSE.
## notch went outside hinges. Try setting notch=FALSE.
```

```
# produce summary statistics
pp_check(object = fit_prot1_post2b, "stat_2d")
```

```
## Using all posterior draws for ppc type 'stat_2d' by default.
```

```
# produce intervals
pp_check(object = fit_prot1_post2b, "intervals")
```

```
## Using all posterior draws for ppc type 'intervals' by default.
```

```
# predctive errors
pp_check(object = fit_prot1_post2b, "error_hist")
```

```
## Using 10 posterior draws for ppc type 'error_hist' by default.
```

```
## `stat_bin()` using `bins = 30`. Pick better value with `binwidth`.
```

# 4 Random effects for replicate structure

Another aspect of the model that we have not considered is the replicate
structure. The replicate structure is a grouping that can be included
as a group-level effect. We can again specify the prior on the standard
deviation of this group-level (random effect).

```
pr <- c(prior(normal(0, 1), class = "b"),
  prior(student_t(3, 0, 1), dpar = "sigma"),
  prior(student_t(3, 0, 0.1), class = "sd"))


fit_prot1_post2c <- brm(
  bf(abundance ~ 0 + type + time + (type:time) + (1|replicate),
     sigma ~ 0 + type + time),
                 data = data,
                 family = gaussian(link = "identity"),
                 sample_prior = "no",
                 prior = pr,
                 control = list(adapt_delta = 0.99),
                 save_pars = save_pars(all = TRUE))
```

```
## Compiling Stan program...
```

```
## Start sampling
```

```
## 
## SAMPLING FOR MODEL '831e52a6db1335a136ad8828a8c5a8ef' NOW (CHAIN 1).
## Chain 1: 
## Chain 1: Gradient evaluation took 0 seconds
## Chain 1: 1000 transitions using 10 leapfrog steps per transition would take 0 seconds.
## Chain 1: Adjust your expectations accordingly!
## Chain 1: 
## Chain 1: 
## Chain 1: Iteration:    1 / 2000 [  0%]  (Warmup)
## Chain 1: Iteration:  200 / 2000 [ 10%]  (Warmup)
## Chain 1: Iteration:  400 / 2000 [ 20%]  (Warmup)
## Chain 1: Iteration:  600 / 2000 [ 30%]  (Warmup)
## Chain 1: Iteration:  800 / 2000 [ 40%]  (Warmup)
## Chain 1: Iteration: 1000 / 2000 [ 50%]  (Warmup)
## Chain 1: Iteration: 1001 / 2000 [ 50%]  (Sampling)
## Chain 1: Iteration: 1200 / 2000 [ 60%]  (Sampling)
## Chain 1: Iteration: 1400 / 2000 [ 70%]  (Sampling)
## Chain 1: Iteration: 1600 / 2000 [ 80%]  (Sampling)
## Chain 1: Iteration: 1800 / 2000 [ 90%]  (Sampling)
## Chain 1: Iteration: 2000 / 2000 [100%]  (Sampling)
## Chain 1: 
## Chain 1:  Elapsed Time: 2.8 seconds (Warm-up)
## Chain 1:                1.527 seconds (Sampling)
## Chain 1:                4.327 seconds (Total)
## Chain 1: 
## 
## SAMPLING FOR MODEL '831e52a6db1335a136ad8828a8c5a8ef' NOW (CHAIN 2).
## Chain 2: 
## Chain 2: Gradient evaluation took 0 seconds
## Chain 2: 1000 transitions using 10 leapfrog steps per transition would take 0 seconds.
## Chain 2: Adjust your expectations accordingly!
## Chain 2: 
## Chain 2: 
## Chain 2: Iteration:    1 / 2000 [  0%]  (Warmup)
## Chain 2: Iteration:  200 / 2000 [ 10%]  (Warmup)
## Chain 2: Iteration:  400 / 2000 [ 20%]  (Warmup)
## Chain 2: Iteration:  600 / 2000 [ 30%]  (Warmup)
## Chain 2: Iteration:  800 / 2000 [ 40%]  (Warmup)
## Chain 2: Iteration: 1000 / 2000 [ 50%]  (Warmup)
## Chain 2: Iteration: 1001 / 2000 [ 50%]  (Sampling)
## Chain 2: Iteration: 1200 / 2000 [ 60%]  (Sampling)
## Chain 2: Iteration: 1400 / 2000 [ 70%]  (Sampling)
## Chain 2: Iteration: 1600 / 2000 [ 80%]  (Sampling)
## Chain 2: Iteration: 1800 / 2000 [ 90%]  (Sampling)
## Chain 2: Iteration: 2000 / 2000 [100%]  (Sampling)
## Chain 2: 
## Chain 2:  Elapsed Time: 1.967 seconds (Warm-up)
## Chain 2:                2.1 seconds (Sampling)
## Chain 2:                4.067 seconds (Total)
## Chain 2: 
## 
## SAMPLING FOR MODEL '831e52a6db1335a136ad8828a8c5a8ef' NOW (CHAIN 3).
## Chain 3: 
## Chain 3: Gradient evaluation took 0 seconds
## Chain 3: 1000 transitions using 10 leapfrog steps per transition would take 0 seconds.
## Chain 3: Adjust your expectations accordingly!
## Chain 3: 
## Chain 3: 
## Chain 3: Iteration:    1 / 2000 [  0%]  (Warmup)
## Chain 3: Iteration:  200 / 2000 [ 10%]  (Warmup)
## Chain 3: Iteration:  400 / 2000 [ 20%]  (Warmup)
## Chain 3: Iteration:  600 / 2000 [ 30%]  (Warmup)
## Chain 3: Iteration:  800 / 2000 [ 40%]  (Warmup)
## Chain 3: Iteration: 1000 / 2000 [ 50%]  (Warmup)
## Chain 3: Iteration: 1001 / 2000 [ 50%]  (Sampling)
## Chain 3: Iteration: 1200 / 2000 [ 60%]  (Sampling)
## Chain 3: Iteration: 1400 / 2000 [ 70%]  (Sampling)
## Chain 3: Iteration: 1600 / 2000 [ 80%]  (Sampling)
## Chain 3: Iteration: 1800 / 2000 [ 90%]  (Sampling)
## Chain 3: Iteration: 2000 / 2000 [100%]  (Sampling)
## Chain 3: 
## Chain 3:  Elapsed Time: 2.405 seconds (Warm-up)
## Chain 3:                2.263 seconds (Sampling)
## Chain 3:                4.668 seconds (Total)
## Chain 3: 
## 
## SAMPLING FOR MODEL '831e52a6db1335a136ad8828a8c5a8ef' NOW (CHAIN 4).
## Chain 4: 
## Chain 4: Gradient evaluation took 0 seconds
## Chain 4: 1000 transitions using 10 leapfrog steps per transition would take 0 seconds.
## Chain 4: Adjust your expectations accordingly!
## Chain 4: 
## Chain 4: 
## Chain 4: Iteration:    1 / 2000 [  0%]  (Warmup)
## Chain 4: Iteration:  200 / 2000 [ 10%]  (Warmup)
## Chain 4: Iteration:  400 / 2000 [ 20%]  (Warmup)
## Chain 4: Iteration:  600 / 2000 [ 30%]  (Warmup)
## Chain 4: Iteration:  800 / 2000 [ 40%]  (Warmup)
## Chain 4: Iteration: 1000 / 2000 [ 50%]  (Warmup)
## Chain 4: Iteration: 1001 / 2000 [ 50%]  (Sampling)
## Chain 4: Iteration: 1200 / 2000 [ 60%]  (Sampling)
## Chain 4: Iteration: 1400 / 2000 [ 70%]  (Sampling)
## Chain 4: Iteration: 1600 / 2000 [ 80%]  (Sampling)
## Chain 4: Iteration: 1800 / 2000 [ 90%]  (Sampling)
## Chain 4: Iteration: 2000 / 2000 [100%]  (Sampling)
## Chain 4: 
## Chain 4:  Elapsed Time: 2.333 seconds (Warm-up)
## Chain 4:                1.778 seconds (Sampling)
## Chain 4:                4.111 seconds (Total)
## Chain 4:
```

```
## Warning: There were 4 divergent transitions after warmup. See
## http://mc-stan.org/misc/warnings.html#divergent-transitions-after-warmup
## to find out why this is a problem and how to eliminate them.
```

```
## Warning: Examine the pairs() plot to diagnose sampling problems
```

```
plot(fit_prot1_post2c, ask = FALSE)
```

```
summary(fit_prot1_post2c)
```

```
## Warning: There were 4 divergent transitions after warmup. Increasing adapt_delta
## above 0.99 may help. See http://mc-stan.org/misc/warnings.html#divergent-
## transitions-after-warmup
```

```
##  Family: gaussian 
##   Links: mu = identity; sigma = log 
## Formula: abundance ~ 0 + type + time + (type:time) + (1 | replicate) 
##          sigma ~ 0 + type + time
##    Data: data (Number of observations: 20) 
##   Draws: 4 chains, each with iter = 2000; warmup = 1000; thin = 1;
##          total post-warmup draws = 4000
## 
## Group-Level Effects: 
## ~replicate (Number of levels: 4) 
##               Estimate Est.Error l-95% CI u-95% CI Rhat Bulk_ESS Tail_ESS
## sd(Intercept)     0.06      0.05     0.00     0.17 1.00      833     1006
## 
## Population-Level Effects: 
##                  Estimate Est.Error l-95% CI u-95% CI Rhat Bulk_ESS Tail_ESS
## typeoops             0.10      0.07    -0.06     0.24 1.00     1434     1058
## typetotal            0.31      0.08     0.16     0.47 1.00     1288     1176
## time23              -0.28      0.13    -0.52    -0.01 1.01     1877     1191
## time6                0.05      0.07    -0.07     0.19 1.00     2038     1442
## typetotal:time23     0.13      0.19    -0.25     0.50 1.00     1659     1616
## typetotal:time6     -0.23      0.09    -0.42    -0.04 1.00     1405     1299
## sigma_typeoops      -2.69      0.68    -4.03    -1.31 1.00      830     1327
## sigma_typetotal     -2.56      0.59    -3.61    -1.30 1.00     1252     1626
## sigma_time23         0.79      0.62    -0.39     2.00 1.00     1096     1600
## sigma_time6         -0.50      0.58    -1.71     0.56 1.00     1365     1749
## 
## Draws were sampled using sampling(NUTS). For each parameter, Bulk_ESS
## and Tail_ESS are effective sample size measures, and Rhat is the potential
## scale reduction factor on split chains (at convergence, Rhat = 1).
```

There are a few divergent transitions suggesting that some aspects of the model
might be computational unfaithful. Given that there are only few we do not worry
about this here. We continue to perform posterior predictive checks.

```
# compute kernel density estimate of data and simulated data and plot
pp_check(object = fit_prot1_post2c, "dens_overlay")
```

```
## Using 10 posterior draws for ppc type 'dens_overlay' by default.
```

```
# plot data in a histogram to see distributions
pp_check(object = fit_prot1_post2c, "hist")
```

```
## Using 10 posterior draws for ppc type 'hist' by default.
```

```
## `stat_bin()` using `bins = 30`. Pick better value with `binwidth`.
```

```
# plot data in a boxplot to see distribtuions
pp_check(object = fit_prot1_post2c, "boxplot")
```

```
## Using 10 posterior draws for ppc type 'boxplot' by default.
```

```
## notch went outside hinges. Try setting notch=FALSE.
## notch went outside hinges. Try setting notch=FALSE.
## notch went outside hinges. Try setting notch=FALSE.
## notch went outside hinges. Try setting notch=FALSE.
```

```
# produce summary statistics
pp_check(object = fit_prot1_post2c, "stat_2d")
```

```
## Using all posterior draws for ppc type 'stat_2d' by default.
```

```
# produce intervals
pp_check(object = fit_prot1_post2c, "intervals")
```

```
## Using all posterior draws for ppc type 'intervals' by default.
```

```
# predctive errors
pp_check(object = fit_prot1_post2c, "error_hist")
```

```
## Using 10 posterior draws for ppc type 'error_hist' by default.
```

```
## `stat_bin()` using `bins = 30`. Pick better value with `binwidth`.
```

## model selection with posterior probabilities

The posterior predictive checks look good. However, is this really a better
model than the previous models. Using probability, we can actually quantify
the extent to which this model is preferred. The following code chunk
using bridge sampling to estimate the posterior model probabilities.

```
post_prob(fit_prot1_post2b,
          fit_prot1_post2c,
          prior_prob = c(0.5,0.5),
          model_names = c("no random effect", "random effect"))
```

```
## Iteration: 1
## Iteration: 2
## Iteration: 3
## Iteration: 4
## Iteration: 5
## Iteration: 6
## Iteration: 1
## Iteration: 2
## Iteration: 3
## Iteration: 4
## Iteration: 5
## Iteration: 6
```

```
## no random effect    random effect 
##        0.4118754        0.5881246
```

The computation here suggests that the model with random effects is preferred
but the probability is only about \(0.6\).

## 4.1 Model selection with out-of-sample posterior predictive inference

We can also use predictive cross-validation checks to see if this is a better model. We use
leave-one-out (loo) cross-validation with log predictive density as the utility
function to evaluate the models.

```
loo_compare(loo(fit_prot1_post2b, moment_match = TRUE, reloo = TRUE),
            loo(fit_prot1_post2c, moment_match = TRUE, reloo = TRUE))
```

```
## Warning: Some Pareto k diagnostic values are slightly high. See help('pareto-k-diagnostic') for details.
```

```
## No problematic observations found. Returning the original 'loo' object.
```

```
## Warning: Some Pareto k diagnostic values are slightly high. See help('pareto-k-diagnostic') for details.
```

```
## No problematic observations found. Returning the original 'loo' object.
```

```
##                  elpd_diff se_diff
## fit_prot1_post2c  0.0       0.0   
## fit_prot1_post2b -1.9       1.4
```

Here we can see that according to loo-cv that the model with random effects
is preferred.

## 4.2 Further random effects

We can now add a random effect according to the TMT tag that
was used.

```
pr <- c(prior(normal(0, 1), class = "b"),
  prior(student_t(3, 0, 1), dpar = "sigma"),
  prior(student_t(3, 0, 0.1), class = "sd"))


fit_prot1_post2d <- brm(bf(abundance ~ 0 + type + time + (type:time) + 
                             (1|replicate) + (1|tag),
                     sigma ~ 0 + type + time),
                 data = data,
                 family = gaussian(link = "identity"),
                 sample_prior = "no",
                 prior = pr,
                 control = list(adapt_delta = 0.99),
                 save_pars = save_pars(all = TRUE))
```

```
## Compiling Stan program...
```

```
## Start sampling
```

```
## 
## SAMPLING FOR MODEL 'dac451c9a8e3ef36209818d29048761e' NOW (CHAIN 1).
## Chain 1: 
## Chain 1: Gradient evaluation took 0 seconds
## Chain 1: 1000 transitions using 10 leapfrog steps per transition would take 0 seconds.
## Chain 1: Adjust your expectations accordingly!
## Chain 1: 
## Chain 1: 
## Chain 1: Iteration:    1 / 2000 [  0%]  (Warmup)
## Chain 1: Iteration:  200 / 2000 [ 10%]  (Warmup)
## Chain 1: Iteration:  400 / 2000 [ 20%]  (Warmup)
## Chain 1: Iteration:  600 / 2000 [ 30%]  (Warmup)
## Chain 1: Iteration:  800 / 2000 [ 40%]  (Warmup)
## Chain 1: Iteration: 1000 / 2000 [ 50%]  (Warmup)
## Chain 1: Iteration: 1001 / 2000 [ 50%]  (Sampling)
## Chain 1: Iteration: 1200 / 2000 [ 60%]  (Sampling)
## Chain 1: Iteration: 1400 / 2000 [ 70%]  (Sampling)
## Chain 1: Iteration: 1600 / 2000 [ 80%]  (Sampling)
## Chain 1: Iteration: 1800 / 2000 [ 90%]  (Sampling)
## Chain 1: Iteration: 2000 / 2000 [100%]  (Sampling)
## Chain 1: 
## Chain 1:  Elapsed Time: 4.109 seconds (Warm-up)
## Chain 1:                3.496 seconds (Sampling)
## Chain 1:                7.605 seconds (Total)
## Chain 1: 
## 
## SAMPLING FOR MODEL 'dac451c9a8e3ef36209818d29048761e' NOW (CHAIN 2).
## Chain 2: 
## Chain 2: Gradient evaluation took 0 seconds
## Chain 2: 1000 transitions using 10 leapfrog steps per transition would take 0 seconds.
## Chain 2: Adjust your expectations accordingly!
## Chain 2: 
## Chain 2: 
## Chain 2: Iteration:    1 / 2000 [  0%]  (Warmup)
## Chain 2: Iteration:  200 / 2000 [ 10%]  (Warmup)
## Chain 2: Iteration:  400 / 2000 [ 20%]  (Warmup)
## Chain 2: Iteration:  600 / 2000 [ 30%]  (Warmup)
## Chain 2: Iteration:  800 / 2000 [ 40%]  (Warmup)
## Chain 2: Iteration: 1000 / 2000 [ 50%]  (Warmup)
## Chain 2: Iteration: 1001 / 2000 [ 50%]  (Sampling)
## Chain 2: Iteration: 1200 / 2000 [ 60%]  (Sampling)
## Chain 2: Iteration: 1400 / 2000 [ 70%]  (Sampling)
## Chain 2: Iteration: 1600 / 2000 [ 80%]  (Sampling)
## Chain 2: Iteration: 1800 / 2000 [ 90%]  (Sampling)
## Chain 2: Iteration: 2000 / 2000 [100%]  (Sampling)
## Chain 2: 
## Chain 2:  Elapsed Time: 4.266 seconds (Warm-up)
## Chain 2:                2.718 seconds (Sampling)
## Chain 2:                6.984 seconds (Total)
## Chain 2: 
## 
## SAMPLING FOR MODEL 'dac451c9a8e3ef36209818d29048761e' NOW (CHAIN 3).
## Chain 3: 
## Chain 3: Gradient evaluation took 0 seconds
## Chain 3: 1000 transitions using 10 leapfrog steps per transition would take 0 seconds.
## Chain 3: Adjust your expectations accordingly!
## Chain 3: 
## Chain 3: 
## Chain 3: Iteration:    1 / 2000 [  0%]  (Warmup)
## Chain 3: Iteration:  200 / 2000 [ 10%]  (Warmup)
## Chain 3: Iteration:  400 / 2000 [ 20%]  (Warmup)
## Chain 3: Iteration:  600 / 2000 [ 30%]  (Warmup)
## Chain 3: Iteration:  800 / 2000 [ 40%]  (Warmup)
## Chain 3: Iteration: 1000 / 2000 [ 50%]  (Warmup)
## Chain 3: Iteration: 1001 / 2000 [ 50%]  (Sampling)
## Chain 3: Iteration: 1200 / 2000 [ 60%]  (Sampling)
## Chain 3: Iteration: 1400 / 2000 [ 70%]  (Sampling)
## Chain 3: Iteration: 1600 / 2000 [ 80%]  (Sampling)
## Chain 3: Iteration: 1800 / 2000 [ 90%]  (Sampling)
## Chain 3: Iteration: 2000 / 2000 [100%]  (Sampling)
## Chain 3: 
## Chain 3:  Elapsed Time: 6.107 seconds (Warm-up)
## Chain 3:                2.018 seconds (Sampling)
## Chain 3:                8.125 seconds (Total)
## Chain 3: 
## 
## SAMPLING FOR MODEL 'dac451c9a8e3ef36209818d29048761e' NOW (CHAIN 4).
## Chain 4: 
## Chain 4: Gradient evaluation took 0 seconds
## Chain 4: 1000 transitions using 10 leapfrog steps per transition would take 0 seconds.
## Chain 4: Adjust your expectations accordingly!
## Chain 4: 
## Chain 4: 
## Chain 4: Iteration:    1 / 2000 [  0%]  (Warmup)
## Chain 4: Iteration:  200 / 2000 [ 10%]  (Warmup)
## Chain 4: Iteration:  400 / 2000 [ 20%]  (Warmup)
## Chain 4: Iteration:  600 / 2000 [ 30%]  (Warmup)
## Chain 4: Iteration:  800 / 2000 [ 40%]  (Warmup)
## Chain 4: Iteration: 1000 / 2000 [ 50%]  (Warmup)
## Chain 4: Iteration: 1001 / 2000 [ 50%]  (Sampling)
## Chain 4: Iteration: 1200 / 2000 [ 60%]  (Sampling)
## Chain 4: Iteration: 1400 / 2000 [ 70%]  (Sampling)
## Chain 4: Iteration: 1600 / 2000 [ 80%]  (Sampling)
## Chain 4: Iteration: 1800 / 2000 [ 90%]  (Sampling)
## Chain 4: Iteration: 2000 / 2000 [100%]  (Sampling)
## Chain 4: 
## Chain 4:  Elapsed Time: 4.605 seconds (Warm-up)
## Chain 4:                3.745 seconds (Sampling)
## Chain 4:                8.35 seconds (Total)
## Chain 4:
```

```
## Warning: There were 8 divergent transitions after warmup. See
## http://mc-stan.org/misc/warnings.html#divergent-transitions-after-warmup
## to find out why this is a problem and how to eliminate them.
```

```
## Warning: Examine the pairs() plot to diagnose sampling problems
```

```
plot(fit_prot1_post2d, ask = FALSE)
```

```
summary(fit_prot1_post2d)
```

```
## Warning: There were 8 divergent transitions after warmup. Increasing adapt_delta
## above 0.99 may help. See http://mc-stan.org/misc/warnings.html#divergent-
## transitions-after-warmup
```

```
##  Family: gaussian 
##   Links: mu = identity; sigma = log 
## Formula: abundance ~ 0 + type + time + (type:time) + (1 | replicate) + (1 | tag) 
##          sigma ~ 0 + type + time
##    Data: data (Number of observations: 20) 
##   Draws: 4 chains, each with iter = 2000; warmup = 1000; thin = 1;
##          total post-warmup draws = 4000
## 
## Group-Level Effects: 
## ~replicate (Number of levels: 4) 
##               Estimate Est.Error l-95% CI u-95% CI Rhat Bulk_ESS Tail_ESS
## sd(Intercept)     0.05      0.04     0.00     0.15 1.00     1400     1839
## 
## ~tag (Number of levels: 10) 
##               Estimate Est.Error l-95% CI u-95% CI Rhat Bulk_ESS Tail_ESS
## sd(Intercept)     0.03      0.03     0.00     0.10 1.00     1339     1446
## 
## Population-Level Effects: 
##                  Estimate Est.Error l-95% CI u-95% CI Rhat Bulk_ESS Tail_ESS
## typeoops             0.09      0.08    -0.08     0.24 1.00     1730     1524
## typetotal            0.31      0.08     0.15     0.47 1.00     2148     1524
## time23              -0.27      0.14    -0.54     0.01 1.00     2265     1675
## time6                0.06      0.08    -0.09     0.24 1.00     1897     1462
## typetotal:time23     0.12      0.19    -0.27     0.50 1.00     2247     1982
## typetotal:time6     -0.24      0.10    -0.44    -0.03 1.00     1854     1392
## sigma_typeoops      -2.52      0.69    -3.90    -1.18 1.00     1156     1440
## sigma_typetotal     -2.50      0.63    -3.68    -1.20 1.00     1173     1656
## sigma_time23         0.67      0.61    -0.51     1.90 1.00     1574     2211
## sigma_time6         -0.62      0.60    -1.86     0.46 1.00     1778     2010
## 
## Draws were sampled using sampling(NUTS). For each parameter, Bulk_ESS
## and Tail_ESS are effective sample size measures, and Rhat is the potential
## scale reduction factor on split chains (at convergence, Rhat = 1).
```

We note that again this model has a few divergent transitions, suggesting
that again this model is becoming difficult to fit. Whilst there are few such
divergences we are not concerned here. Again, we can examine posterior
predictive checks, posterior model probabilities and loo evaluation of
the previous models.

```
# compute kernel density estimate of data and simulated data and plot
pp_check(object = fit_prot1_post2d, "dens_overlay")
```

```
## Using 10 posterior draws for ppc type 'dens_overlay' by default.
```

```
# plot data in a histogram to see distributions
pp_check(object = fit_prot1_post2d, "hist")
```

```
## Using 10 posterior draws for ppc type 'hist' by default.
```

```
## `stat_bin()` using `bins = 30`. Pick better value with `binwidth`.
```

```
# plot data in a boxplot to see distribtuions
pp_check(object = fit_prot1_post2d, "boxplot")
```

```
## Using 10 posterior draws for ppc type 'boxplot' by default.
```

```
## notch went outside hinges. Try setting notch=FALSE.
## notch went outside hinges. Try setting notch=FALSE.
## notch went outside hinges. Try setting notch=FALSE.
## notch went outside hinges. Try setting notch=FALSE.
## notch went outside hinges. Try setting notch=FALSE.
```

```
# produce summary statistics
pp_check(object = fit_prot1_post2d, "stat_2d")
```

```
## Using all posterior draws for ppc type 'stat_2d' by default.
```

```
# produce intervals
pp_check(object = fit_prot1_post2d, "intervals")
```

```
## Using all posterior draws for ppc type 'intervals' by default.
```

```
# predctive errors
pp_check(object = fit_prot1_post2d, "error_hist")
```

```
## Using 10 posterior draws for ppc type 'error_hist' by default.
```

```
## `stat_bin()` using `bins = 30`. Pick better value with `binwidth`.
```

```
post_prob(fit_prot1_post2b,
          fit_prot1_post2c,
          fit_prot1_post2d,
          prior_prob = c(1/3,1/3, 1/3),
          model_names = c("no random effect",
                          "random effect",
                          "two random effects"))
```

```
## Iteration: 1
## Iteration: 2
## Iteration: 3
## Iteration: 4
## Iteration: 5
## Iteration: 6
## Iteration: 1
## Iteration: 2
## Iteration: 3
## Iteration: 4
## Iteration: 5
## Iteration: 6
## Iteration: 7
## Iteration: 1
## Iteration: 2
## Iteration: 3
## Iteration: 4
## Iteration: 5
## Iteration: 6
## Iteration: 7
```

```
##   no random effect      random effect two random effects 
##          0.3543369          0.4753413          0.1703218
```

```
loo_compare(loo(fit_prot1_post2b, moment_match = TRUE, reloo = TRUE),
            loo(fit_prot1_post2c, moment_match = TRUE, reloo = TRUE),
            loo(fit_prot1_post2d, moment_match = TRUE, reloo = TRUE))
```

```
## Warning: Some Pareto k diagnostic values are slightly high. See help('pareto-k-diagnostic') for details.
```

```
## No problematic observations found. Returning the original 'loo' object.
```

```
## Warning: Some Pareto k diagnostic values are slightly high. See help('pareto-k-diagnostic') for details.
```

```
## No problematic observations found. Returning the original 'loo' object.
```

```
## Warning: Some Pareto k diagnostic values are slightly high. See help('pareto-k-diagnostic') for details.
```

```
## No problematic observations found. Returning the original 'loo' object.
```

```
##                  elpd_diff se_diff
## fit_prot1_post2d  0.0       0.0   
## fit_prot1_post2c -0.4       1.0   
## fit_prot1_post2b -2.4       1.6
```

From the above analysis, we can see that when using posterior model probabilities
the model with random effect according to replicates is preferred and the model
using random effects for replicates and tags the least preferred model. However,
when using LOO-CV the preferred model is the model with random effects for tags
and replicates followed shortly by the other model with random effects.

The question now is with which model do we progress. Ultimately, this will come
down to what we want the model to do. However, it appears that there
is not a clear case for the more complex random effects model and so favoring
simplicity the simpler random effects model could be justified. If users
were so inclined they could also average these models using the posterior model
probabilities as weights.

# 5 Using uncertainty

We return to our original question of whether there is a change in proportion
of the total protein bound to RNA. We examine the posterior distribution
from the interactions of interest. We can plot the joint distribution of the
two interactions

```
plot(hexbin::hexbin(posterior_samples(fit_prot1_post2c)[,5:6]),
     colramp = colorRampPalette(brewer.pal(11, "PRGn")),
     xlab = "total:time23h", ylab = "total:time6h")
```

```
## Warning: Method 'posterior_samples' is deprecated. Please see ?as_draws for
## recommended alternatives.
```

```
ggplot(data = data.frame(x = posterior_samples(fit_prot1_post2c)[,5],
       y = posterior_samples(fit_prot1_post2c)[,6]), aes(x = x, y = y)) + geom_hex(bins = 50) + scale_fill_gradient(low = colorRampPalette(brewer.pal(11, "PRGn"))(1), high = colorRampPalette(brewer.pal(11, "PRGn"))(11)) + theme_classic() + xlab("total:time23h") + ylab("total:time6h") + geom_rect(data = data.frame(x=0, y = 0),aes(xmin = -0.1, xmax = 0.1, ymin = -Inf, ymax = Inf),
            alpha = 0.2,
            fill = alpha("orange", 0.2)) + 
geom_rect(data = data.frame(x=0, y = 0),aes(xmin = -Inf, xmax = Inf, ymin = -0.1, ymax = 0.1),
            alpha = 0.2,
            fill = alpha("orange", 0.2)) +  theme(text = element_text(size=20))
```

```
## Warning: Method 'posterior_samples' is deprecated. Please see ?as_draws for
## recommended alternatives.

## Warning: Method 'posterior_samples' is deprecated. Please see ?as_draws for
## recommended alternatives.
```

It is clear that the effects of these interactions are in the opposite
directions for each time. We can compute the probability that these interactions
are < 0 and > 0 respectively. We can also ask about specific effect-sizes we are
interested in. We note that we are slightly more confident that the interaction
at 6h is less than \(-0.1\) in our original analysis likely due to our account
for more variability in our model through the random effect.

```
# Probability greater than 0 interaction at 23h
sum(posterior_samples(fit_prot1_post2c)[,5] > 0)/length(posterior_samples(fit_prot1_post2c)[,5])
```

```
## Warning: Method 'posterior_samples' is deprecated. Please see ?as_draws for
## recommended alternatives.

## Warning: Method 'posterior_samples' is deprecated. Please see ?as_draws for
## recommended alternatives.
```

```
## [1] 0.79525
```

```
# Probability less than 0 interaction at 6h
sum(posterior_samples(fit_prot1_post2c)[,6]  < 0)/length(posterior_samples(fit_prot1_post2c)[,6])
```

```
## Warning: Method 'posterior_samples' is deprecated. Please see ?as_draws for
## recommended alternatives.

## Warning: Method 'posterior_samples' is deprecated. Please see ?as_draws for
## recommended alternatives.
```

```
## [1] 0.98375
```

```
# probability less than -0.1 interaction 6h
sum(posterior_samples(fit_prot1_post2c)[,6]  < -0.1)/length(posterior_samples(fit_prot1_post2c)[,6])
```

```
## Warning: Method 'posterior_samples' is deprecated. Please see ?as_draws for
## recommended alternatives.

## Warning: Method 'posterior_samples' is deprecated. Please see ?as_draws for
## recommended alternatives.
```

```
## [1] 0.938
```

# 6 Conclusion

We have developed a Bayesian mixed-effects model for dynamics oops data.
Demonstrating the clear parts of the workflow. As always, we could further
extend this model but there is only need for complexity if it improves our
inferences or allows us to answer new questions. We could also ask more
questions of these probability distributions and perform model averaging
of several of the different proposed models. We could continue to apply this
model to other proteins. As this case study has shown what we set out to do,
we leave this analysis to further work.
